# STAT6 degradation and ubiquitylated TRIML2 are essential for activation of human oncogenic herpesvirus

**DOI:** 10.1371/journal.ppat.1007416

**Published:** 2018-12-10

**Authors:** Feng Gu, Chong Wang, Fang Wei, Yuyan Wang, Qing Zhu, Ling Ding, Wenjia Xu, Caixia Zhu, Cankun Cai, Zhikang Qian, Zhenghong Yuan, Erle Robertson, Qiliang Cai

**Affiliations:** 1 MOE& MOH Key Laboratory of Medical Molecular Virology, School of Basic Medicine, Shanghai Medical College, Fudan University, Shanghai, P. R. China; 2 ShengYushou Center of Cell Biology and Immunology, School of Life Sciences and Biotechnology, Shanghai Jiao Tong University, Shanghai, P. R. China; 3 Unit of Herpesvirus and Molecular Virology, Key Laboratory of Molecular Virology &Immunology, Institute Pasteur of Shanghai, Chinese Academy of Sciences, University of the Chinese Academy of Sciences, Shanghai, P. R. China; 4 Department of Otorhinolaryngology-Head and Neck Surgery, Department of Microbiology, Abramson Comprehensive Cancer Center, Perelman School of Medicine at the University of Pennsylvania, Philadelphia, PA, United States of America; University of Southern California, UNITED STATES

## Abstract

Aberrations in STAT6-mediated signaling are linked to the development of multiple cancer types. Increasing evidence has shown that activation of human oncogenic herpesvirus lytic replication is crucial for viral tumorigenesis. However, the role of STAT6 in herpesvirus lytic replication remains elusive. Here, by using Kaposi’s sarcoma-associated herpesvirus (KSHV) as a model, we revealed that RTA, the master regulator of lytic replication, interacts with STAT6 and promotes lysine 48 (K48) and K63-linked ubiquitylation of STAT6 for degradation via the proteasome and lysosome systems. Moreover, degradation of STAT6 is dramatically associated with the increased ubiquitylated form of tripartite motif family like 2 (TRIML2, a tumor suppressor) for prolonged cell survival and virion production, which is also commonly observed in lytic activation of Epstein-Barr virus, herpes simplex virus 1 and cytomegalovirus. These results suggest that degradation of STAT6 is important for the lytic activation of KSHV and as such, may be an attractive therapeutic target.

## Author summary

STAT6 is a transcriptional factor that plays an important role in the extracellular cytokine and virus-mediated immune response. Extensive studies have revealed that the dysregulation of STAT6 is linked to the pathological features of virus-associated cancers. However, the molecular mechanism of STAT6 regulation by tumor viruses is still unknown. Here, we report that the degradation of STAT6 is induced and required for the lytic activation of human herpesviruses including oncogenic γ-herpesviruses (KSHV and EBV) and α/β-herpesviruses (HSV1 and HCMV). Importantly, this effect is highly dependent on the expression of viral lytic antigens (i.e., RTA in KSHV). This study reveals the central role of STAT6 in controlling the switch from latency to lytic replication of herpesviruses.

## Introduction

The signal transducer and activator of transcription (STAT) family are transcription factors that mediate the transmission of signals of numerous cytokines and growth factors from the cell membrane to the nucleus[1]. To date, there are at least seven identified STAT proteins, named STAT1, 2, 3, 4, 5a, 5b, and 6[2]. Dysregulation of STAT family members results in immune system disorder and cancer. STAT6, an important member of the STAT family, is a key responder to the stimulation of cytokines interleukin 4 (IL-4) and IL-13 in the differentiation of T helper 2 cells[3], and it is also involved in the antiviral immune response[4]. Typically, IL-4/13 binds and induces phosphorylation of the IL-4α receptor at the plasma membrane, which in turn recruits Janus kinase (JAK) and cytosolic STAT6 for phosphorylation[5]. The phosphorylation of STAT6 on tyrosine 641 (Y641) by JAK1 results in dimerization and translocation of STAT6 from the cytoplasm into the nucleus to activate downstream target genes, including IL-4 and CD23 via binding to the consensus DNA sequence TTC(N3/N4)GAA within the promoters[6]. Interestingly, other cytokines, including IL-3/15, IFNα, and PDGF, also activate STAT6 in different cell types[7–10]. In addition, recent studies have shown that primary virus infection can induce STAT6 activation in the endoplasmic reticulum independently of JAK, but it relies on a stimulator of interferon genes and TANK-binding kinase 1 for antiviral innate immunity[4]. This virus-induced STAT6 activation is commonly detected in all cell types, suggesting its fundamental role in the host immune defense against viral infections. Thus, STAT6 mediates the comprehensive regulation of immune signaling in response to both the stimulation of cytokines at the plasma membrane and viral infection in the endoplasmic reticulum.

In addition to playing a key role in the regulation of extracellular cytokines and viral infection, STAT6 is also involved in the immune response against nematode infection and the development of allergic inflammatory disease (i.e., airway hyper responsiveness or eosinophilic inflammation)[11,12]. Increasing evidence indicates that STAT6 also contributes to tumor formation, and STAT6 activation is often observed in many malignancies, including follicular lymphoma, colon cancer, and classical Hodgkin lymphoma[13–16]. STAT6 deficiency not only affects macrophage development, but it also impairs major histocompatibility complex (MHC) class II expression and nitric oxide production, which results in higher susceptibility to viral infection[4].

Kaposi’s sarcoma-associated herpesvirus (KSHV), also known as human herpesvirus 8, was discovered by Chang and Moore in 1994 by representational difference analysis[17]. As an oncogenic γ-herpesvirus, KSHV is highly homologous with Epstein-Barr virus (EBV, the first identified tumor virus in humans), and has been documented as the etiological agent for Kaposi’s sarcoma (KS)[18], primary effusion lymphoma (PEL)[19], and multicentric Castleman disease (MCD)[20]. Similar to other herpesviruses, KSHV also undergoes two life cycle phases: latency and lytic replication. During latency, only a limited number of viral transcripts are expressed and the viral genome persists as a circular episome tethered to the host chromatin through the latency-associated nuclear antigen (LANA)[21]. The lytic cycle is characterized by the expression of most viral genes in an orderly fashion (immediate early, early, and late), and eventually the production of infectious virion particles. It has been well demonstrated that the switch of KSHV from latency to lytic replication is mediated through the replication and transcription activator (RTA). RTA, encoded by KSHV ORF50, is a conserved viral transcription factor that activates the expression of a series of lytic genes, including K8, ORF57, vIL-6, and vIRF1[22,23]. Similar to other immediate early genes encoded by different herpesviruses (e.g., ZTA in EBV, IE1/2 in HCMV, and ICP0 in HSV1), ectopic expression of RTA is sufficient to disrupt viral latency and activate lytic replication. Apart from genes activated by RTA, many viral and cellular factors, including HDAC1[24,25], poly (ADP-ribose) polymerase 1 (PARP-1)[26], NF-κB[27], K-bZIP[28], and LANA[29], also repress RTA expression. Interestingly, recent studies have demonstrated that RTA possesses ubiquitin E3 ligase activity and can inhibit innate immunity and activate lytic replication by targeting different cellular and viral substrates for proteasome degradation[30].

Regarding the role of STAT6 signaling in herpesvirus infection and mediated pathogenesis, it has been reported that STAT6 can be induced to translocate into the nucleus and activate the immune response by primary infection of herpes simplex virus 1 (HSV-1) or Herpesvirus saimiri (a T-lymphotropic monkey herpesvirus) in different cell types[4,31]. Previous studies from our group and others have shown that KSHV selectively regulates the IL-4/13-induced phosphorylation of STAT6 on Y641 to promote cell proliferation and maintain latency[15,32,33], in addition to inducing nuclear localization and cleavage of STAT6 to block RTA expression during latency[34]. However, whether the expression of STAT6 is inhibited during lytic replication remains unclear.

In this study, we found that RTA interacts with STAT6 to promote K48- and K63-linked ubiquitylation of STAT6 for degradation via the proteasome and lysosomal system. Distinct from other STATs, the degradation of STAT6 is dramatically associated with the increased expression and ubiquitylation of tripartite motif family like 2 (TRIML2, a p53-associated tumor suppressor), which is also commonly observed in other herpesviruses with lytic replication.

## Results

### Expression of STAT6 is reduced during the early infection and lytic cycle of KSHV

**Fig 1:**
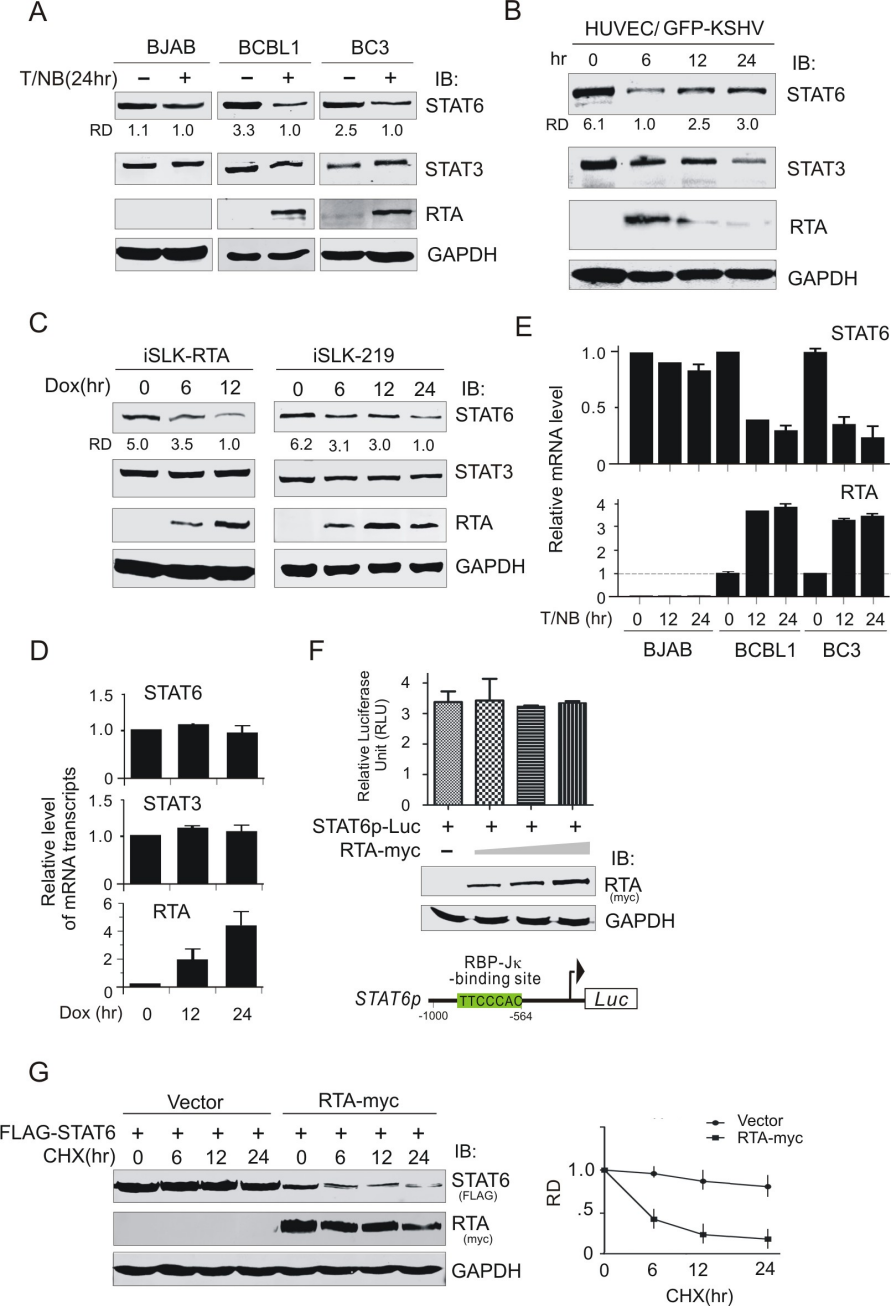
Expression of STAT6 is reduced during KSHV reactivation and early infection. (**A**) Expression of STAT6 but not STAT3 was dramatically reduced during KSHV reactivation. Whole cell lysates from KSHV-infected BCBL1 and BC3 and uninfected BJAB cells individually treated with or without 20 ng/ml of TPA and 1.5mM sodium butyrate (T/NB) for 24 h, were subjected to immunoblotting (IB) with antibodies as indicated in the figure. The relative density (RD) of STAT6 protein band was quantitated. (**B**) Early infection of KSHV reduced STAT6 expression. Whole cell lysate from HUVEC cells with KSHV infection (GFP positive) at different time points, were subjected to immunoblotting (IB) with antibodies as indicated in the figure. (**C**) Doxycycloline (Dox)-induced RTA expression in iSLK-RTA or iSLK-219 cells led to the decreased expression of STAT6 but not of STAT3. Whole cell lysates from the different-induction time points were subjected to immunoblotting (IB) with antibodies as indicated in the figure. (**D**) Quantitative PCR analysis of transcriptional level of STAT6 and STAT3 in iSLK-RTA cells with doxycline induction. (**E**) Quantitative PCR analysis of STAT6 and RTA mRNA transcripts in BJAB, BCBL1 and BC3 cells treated with TPA and sodium butyrate (T/NB) for 0, 12 and 24 h. The relative level of mRNA transcript was present. Beta actin was used as internal control. (**F**) Reporter assays of STAT6 promoter. HEK293 cells co-transfected STAT6 promoter-driven luciferase reporter with different dosage RTA (0, 1, 5, 10μg) were subjected to reporter assay. Relative firefly luciferase unit (RLU) normalization with Renilla activity was analyzed. Data is presented as means±SD of three independent experiments. The expression of exogenous RTA was verified by immunoblotting assays and shown in the middle panel. Schematic of putative RBP-Jκ-binding sites within STAT6 promoter is shown at the bottom panel. (**G**) RTA reduces the protein stability of STAT6. HEK293T cells were co-transfected by FLAG-STAT6 with RTA-myc or vector alone. At 36 h post-transfection, cells were treated with Cycloheximide (CHX) 200μg/ml for the indicated time before harvesting and lysing for immunoblotting. The relative density (RD) of protein level of STAT6 is quantified based on triplicate experiments and shown at the bottom panel.

Our previous studies demonstrated that KSHV induces STAT6 nuclear location and cleavage for maintaining latency[34]. Interestingly, we also observed that the protein level of STAT6 was downregulated when KSHV-infected PEL cells were treated with tetradecanoyl phorbol acetate (TPA) and sodium butyrate. To determine if the inhibition of STAT6 expression is specifically due to reactivation of the KSHV lytic cycle, we treated KSHV-negative BJAB cells, KSHV-positive BCBL1 cells, and BC3 B-lymphoma cells with TPA and sodium butyrate for 24 h, followed by Western blot analysis of STAT6, STAT3, and RTA (a master reactivator from latency to the lytic cycle that is highly expressed during the KSHV lytic cycle). The results showed that the protein levels of STAT6 were dramatically reduced in both BCBL1 and BC3 cells (in which RTA was markedly upregulated upon TPA and sodium butyrate treatment for KSHV reactivation of the lytic cycle) instead of BJAB cells (Fig 1A). In contrast, the protein level of STAT3 remained unchanged in both KSHV-negative and KSHV-positive B-lymphoma cells, showing that the inhibitory effects of lytic cycle reactivation on STAT6 were specific. Since KSHV also undergoes lytic replication in the early stages of primary infection, we examined the protein level of STAT6 in human umbilical vein endothelial cells (HUVECs) with *de novo* infection of KSHV at different time points. The data showed that the protein level of STAT6 was dramatically reduced in the early stage (6 h) of primary infection, but gradually recovered with longer infection, which is compromised with the expression of RTA decreased (Fig 1B), albeit the protein levels of STAT3 were also reduced to some extent These results further support the notion that the reduced expression of STAT6 in the KSHV lytic cycle is due to RTA expression. To further confirm this hypothesis, we examined the protein levels of STAT6 and STAT3 in RTA-inducible iSLK cells or KSHV genome-carrying iSLK-219 cells with or without doxycline (Dox) treatment. The results showed that only STAT6 instead of STAT3 was reduced by RTA in a dose-dependent manner (Fig 1C).

### RTA induces STAT6 degradation via the proteasome and lysosome

**Fig 2:**
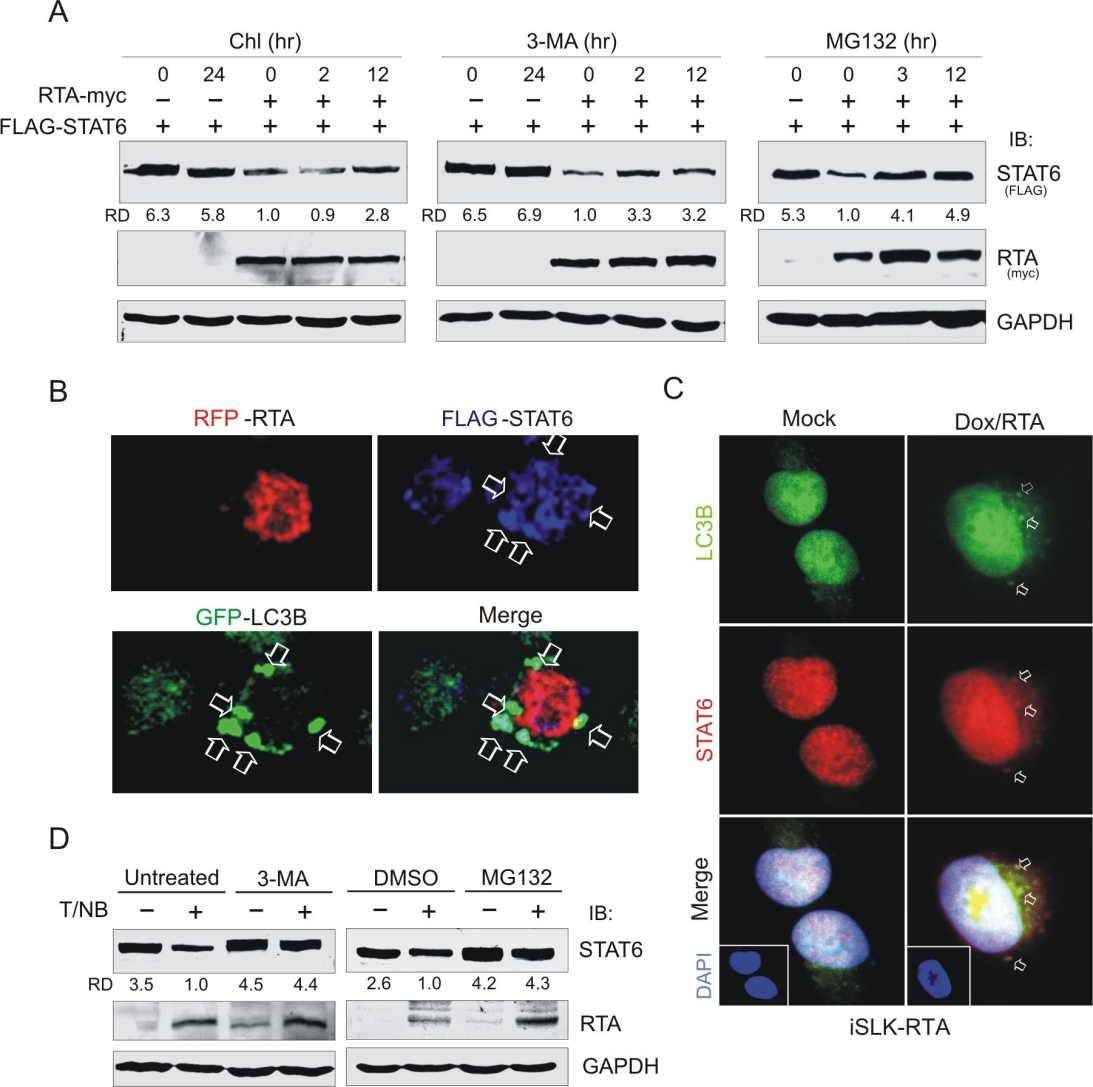
RTA-induced degradation of STAT6 is dependent on activities of the lysosome and proteasome. (**A**) 293T cells were transfected with exogenous FLAG-STAT6 in the presence or absence of myc-tagged RTA. At 24 h post-transfection, cells were individually subjected to treatment with lysosomal inhibitor Chloroquine (Chl) and 3-Methyladenine (3-MA), or proteasomal inhibitor MG-132, followed by immunoblotting with antibodies as indicated in the figure. The relative density (RD) of STAT6 protein band was quantitated. (**B**) RTA induced exogenous STAT6 co-localization with LC3B. 293T cells transfected with FLAG-STAT6, RFP-RTA and GFP-LC3B were subjected to immunofluorescent confocal assays with GFP (green), RFP (red) and FLAG (blue) antibody. Arrow indicates the co-localization of STAT6 with LC3B. (**C**) RTA induced endogenous STAT6 co-localization with LC3B. iSLK-RTA cells treated with or without doxycycloline (Dox) for 12 h, were subjected to immunofluorescent assays with antibodies against STAT6 or LC3B. Nuclei were stained with DAPI. (**D**) Inhibitors of lysosome and proteasome activity blocked RTA-induced STAT6 degradation during lytic reactivation. BCBL1 cells were induced with or without TPA and sodium butyrate (T/NB) for 12 h, followed by individually treatment with 3-MA, MG-132, or DMSO for 3 hours, and subjected to immunoblotting assays as indicated in figure.

To determine if the inhibition of STAT6 expression by RTA is due to regulation at the transcriptional or translational level, the mRNA level of STAT6 in iSLK-RTA cells treated with Dox or PEL cells treated with TPA and sodium butyrate for the induction of RTA expression was individually quantified by quantitative PCR (qPCR). The data showed no significant change in STAT6 transcripts in the presence of RTA in the iSLK-RTA cells (Fig 1D), while treatment with TPA and sodium butyrate reduced the transcription level of STAT6 in both KSHV-positive and negative cells, although it was more significantly in the RTA-expressing PEL cells (Fig 1E). To further confirm that the RTA-mediated inhibition of STAT6 expression did not occur at the transcription level, we generated a luciferase reporter driven by the STAT6 promoter (contains a RBP-Jκ binding site, as previous studies have indicated that RTA interacts with RBP-Jκ to regulate gene transcription), and performed reporter assays by co-transfecting 293 cells with different amounts of RTA. The results showed that STAT6 transcription did not significantly change in the presence of increasing RTA concentrations (Fig 1F). In contrast, immunoblotting results from 293T cells co-transfected with exogenous STAT6 or GFP-NLS and different amount of RTA showed that the protein level of exogenous STAT6 greatly decreased compared to GFP-NLS in the presence of RTA in a dose-dependent manner (S1A Fig). Distinct from RTA, no inhibitory effects on the protein level of exogenous STAT6 were observed upon co-expression with LANA (S1B Fig), suggesting that RTA plays a role in degrading STAT6. Moreover, the fact that the protein stability of exogenous STAT6 was dramatically reduced in the presence of RTA (Fig 1G), further supported the notion that RTA-induced inhibition of STAT6 expression is through regulation at the translational level. To determine if RTA-induced degradation of STAT6 is via the proteasome or lysosomal pathway, we co-expressed exogenous STAT6 with RTA in 293 cells followed by individual treatment with the proteasome inhibitor MG132 or lysosomal inhibitors 3-methyladenine and choloquine for different time points. Fig 2A shows that both the proteasome and lysosomal inhibitors could efficiently inhibit the degradation of STAT6 induced by RTA. Similar RTA-mediated inhibition of exogenous Myd88 by MG132 treatment (which was previously demonstrated and used a parallel positive control) ruled out the potential effects from the transfection system (S2 Fig). To verify that the lysosomal pathway involved RTA-induced degradation of STAT6, 293T cells co-transfected with exogenous STAT6 and RFP-tagged RTA in the presence of GFP-tagged LC3B (a lysosomal marker that efficiently responds to stress as demonstrated in S3 Fig) were subjected to immunofluorescent assays. The dominant punctate foci of exogenous STAT6 co-localized with GFP-LC3B in the presence of RTA were clearly observed (Fig 2B). Similar effect of endogenous STAT6 co-localization with LC3B in the cytoplasm compartment was observed in the RTA-induced iSLK cells (Fig 2C). Interestingly, the inhibitory effects of endogenous STAT6 in BCBL1 cells upon KSHV reactivation were efficiently blocked by both proteasome and lysosomal inhibitors, respectively (Fig 2D), further confirming that RTA-mediated degradation of STAT6 is dependent on both proteasome and lysosomal pathways.

### STAT6 interacts with RTA through its transactivation domain

**Fig 3:**
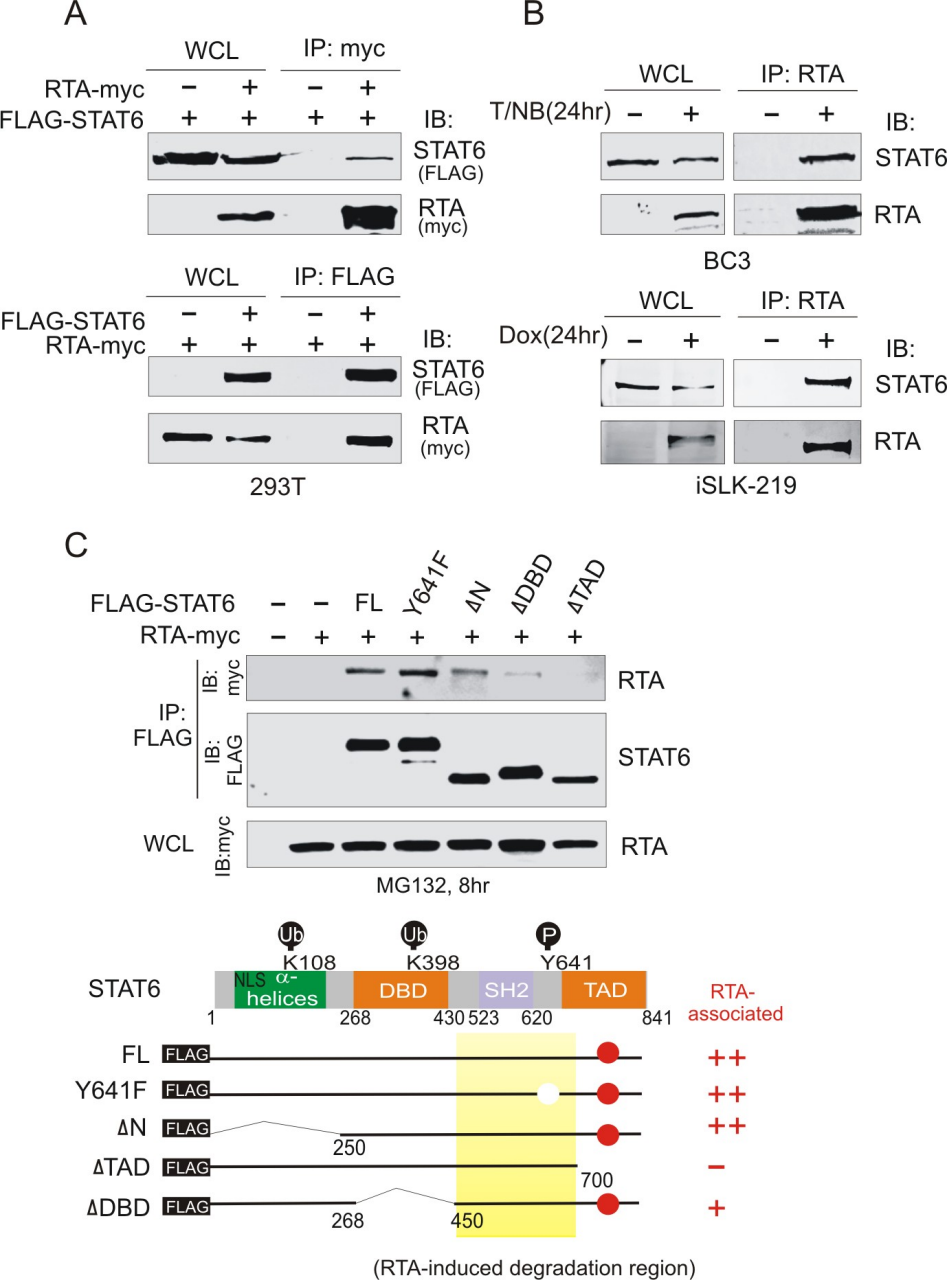
RTA interacts with STAT6. (**A**) Ectopic expression of exogenous STAT6 interacted with RTA. Whole cell lysate (WCL) from 293T cells co-transfected with different combination of FLAG-STAT6 and RTA-myc as indicated, were subjected to immunoprecipitation (IP) followed by immunoblotting (IB) or directly immunoblotting assays as indicated in figure. (**B**) Endogenous STAT6 associated with RTA in KSHV-positive cells with lytic reactivation. Whole cell lysate (WCL) from BC3 cells treated with 20 ng/ml of TPA and 1.5mM sodium butyrate (T/NB) or iSLK-219 cells treated with 2μg/ml doxycycloline (Dox) for 24h were subjected to immunoprecipitation (IP) followed by immunoblotting (IB) or directly immunoblotting assays as indicated in figure. (**C**) RTA associated with the carboxyl terminus of STAT6. 293T cells were transfected with expressing plasmids for 48 h, and the whole cell lysates (WCL) were subjected to immunoprecipitation (IP) followed by immunoblotting (IB) or directly immunoblotting assays as indicated in figure. Schematic of STAT6 amino acid sequence and its truncation mutants were shown at the bottom panels. The red circle indicates the RTA-associated domains. The nuclear localization sequence (NLS), ubiquitylation (Ub) and phosphorylation (P) sites are shown.

To determine if STAT6 associates with RTA, 293T cells were co-transfected with FLAG-tagged STAT6 in the presence or absence of myc-tagged RTA, or myc-tagged RTA in the presence or absence of FLAG-tagged STAT6. The results from the immunoprecipitation (IP) assay showed that STAT6 co-immunoprecipitated with RTA (Fig 3A). Similar results of endogenous STAT6 immunoprecipitating with RTA in BC3 cells and iSLK.219 cells upon reactivation of lytic cycle were observed (Fig 3B), indicating that RTA interacts with STAT6. To identify the STAT6 domain required for interaction with RTA, three truncated STAT6 mutants were generated according to their different functional domains (Fig 3C, bottom panels), followed by IP from cells co-expressing RTA alone or with the different truncation mutants. To avoid the inhibitory effects of RTA on the degradation of STAT6, transfected cells were treated with MG132 before harvesting. As shown in Fig 3C top panels, the deletion of the transactivation domain (ΔTAD) of STAT6 significantly abolished the interaction of STAT6 with RTA compared with full-length STAT6, Y641F mutants, and other two truncated mutants with deletion of the α-helix domain (ΔN) or DNA-binding domain (ΔDBD). These results confirm that RTA interacts with STAT6 through its transactivation domain at the carboxyl terminus.

### RTA-induced degradation of STAT6 is independent of K108, K398 or phosphorylated Y641

**Fig 4:**
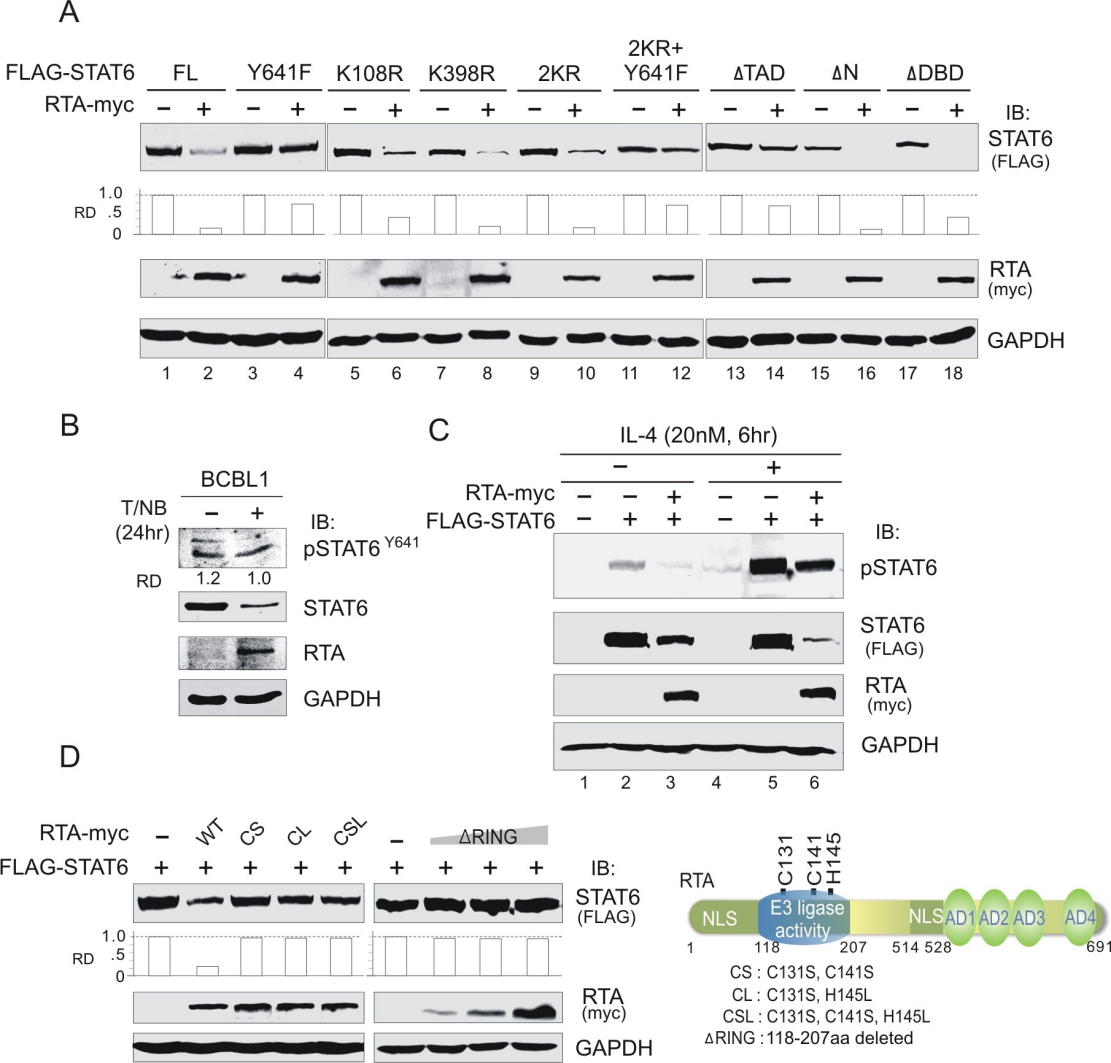
RTA-induced degradation of STAT6 is dependent on the RING-domain of RTA. (**A**) Degradation of STAT6 induced by RTA relied upon its transactivation domain. 293T cells were transfected with expressing plasmids for 48 hours, and the whole cell lysates (WCL) were subjected to immunoprecipitation (IP) followed by immunoblotting (IB) or directly immunoblotting assays as indicated in figure. RD, relative density. (**B**) Endogenous phosphorylated STAT6 on Y641 was not significantly reduced by reactivation of the lytic cycle in PEL cells. BCBL1 cells treated with or without TPA and sodium butyrate (NaB) were subjected to immunoblotting assays as indicated in figure. (**C**) RTA did not block IL-4 induction of STAT6 phosphorylation on Y641. 293T cells were transfected with exogenous FLAG-STAT6 in the presence or absence of myc-tagged RTA. At 24 h post-transfection, cells were subjected to treatment with 20nM IL-4 for 6h before harvesting for immunoblotting with antibodies as indicated in the figure. (**D**) RTA-induced degradation of STAT6 was dependent on its RING-domain. HEK293T cells were transfected STAT6 with FLAG tag in the presence or absence of wild type (WT) RTA with myc tag or its mutants for 48 h, and subjected to immunoblotting (IB) with antibodies as indicated in the figure. The relative density (RD) of STAT6 protein band was quantitated. Brief schematic of RTA amino acid sequence and its mutants is shown on the right panel.

Previous studies have demonstrated that STAT6 degradation is dependent upon ubiquitylation on K108 and K398[35]. To determine if RTA-induced degradation of STAT6 is also dependent on these two lysine residues, we generated a series of STAT6 mutants, including Y641 mutation (a key residue for phosphorylation and activation by cytokine), and co-expressed them in the presence or absence of RTA followed by immunoblotting assays. Interestingly, the results showed that neither lysine 108 (K108R), lysine 398 (K398R), or both (2KR) mutations could efficiently block RTA-induced degradation of STAT6 compared with wild-type (full-length) STAT6 (Fig 4A, compare lane 2 with lanes 6, 8, and 10). In contrast, mutation of tyrosine 641 (Y641F) markedly inhibited the degradation of STAT6 induced by RTA (Fig 4A, compare lane 4 with lane 2), and similar effects with the triple mutant (2KR+Y641F) were observed (Fig 4A, compare lane 12 with lane 2), confirming that Y641 may be required for the RTA-induced degradation of STAT6. In similar assays using truncated mutants of STAT6, mutants with the deletion of the amino terminus (ΔN) or DNA-binding domain (ΔDBD) but containing the SH2 domain (region containing Y641) and TAD (bound to RTA) did not affect the inhibitory effects of RTA on STAT6, whereas the effects were markedly blocked by a deletion mutant of the TAD domain (Fig 4A, compare lane 2 with 18, 16, and 14), further supporting the conclusion that RTA-induced degradation of STAT6 requires the RTA-interacting domain of STAT6.

To determine if the phosphorylation of STAT6 on Y641 contributed to the RTA-induced degradation of STAT6, we detected the protein level of endogenous STAT6 with Y641 phosphorylation in BCBL1 cells by using a specific antibody with or without induction of TPA and sodium butyrate for 24 h. Unexpectedly, as shown in Fig 4B, native STAT6 but not the phosphorylated form of STAT6 on Y641 was significantly reduced in the presence of RTA, ruling out the possibility that RTA-induced degradation of STAT6 is dependent on Y641 phosphorylation. In the ectopic expression of exogenous STAT6 in 293T cells treated with or without IL-4, the results that relative levels of phosphorylated STAT6 induced by IL-4 was not significantly blocked by the presence of RTA (Fig 4C, compare lanes 2, 3 with 5, 6), indicating that phosphorylation on Y641 of STAT6 is not required for RTA to induce STAT6 degradation. The dramatically cytoplasm compartment location of Y641F mutant in the presence or absence of RTA could explain to some extent why Y641F blocks the RTA-induced degradation of STAT6 (S4 Fig).

### RTA promotes K48- and K63-linked ubiquitylation of STAT6

**Fig 5:**
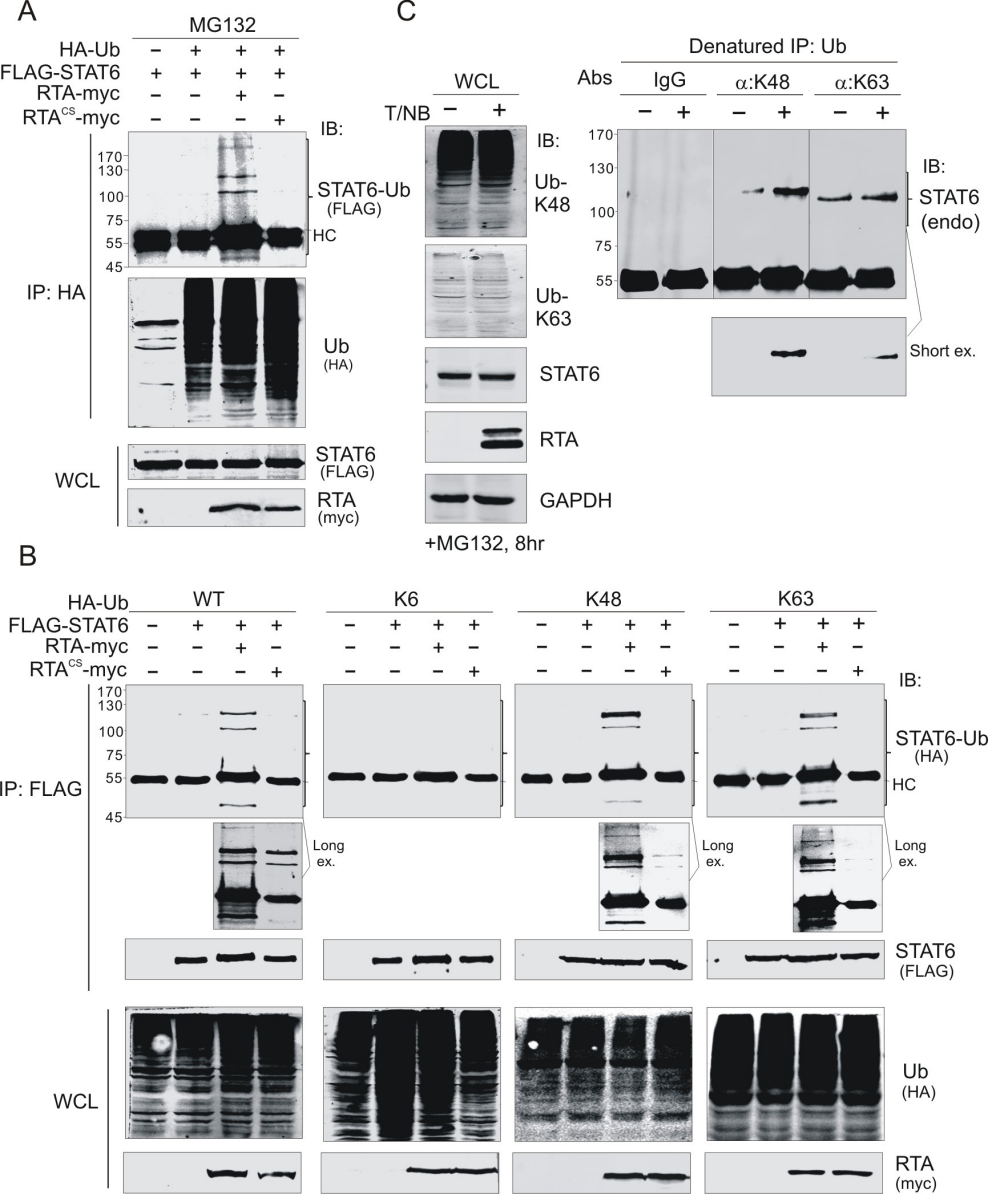
RTA promotes K48 and K63-linked ubiquitylation of STAT6. (**A**) RTA induced STAT6 ubiquitylation. HEK293T cells were co-transfected with different expressing plasmids as indicated. At 36 h post-transfection, cells were treated with proteasomal inhibitor MG132 for 6 h before harvesting and lysing for immunoprecipitation (IP) and immunoblotting (IB). (**B**) K48 and K63-linked ubiquitylation of exogenous STAT6 was induced by RTA. HEK293T cells were co-transfected and treated as in panel A. HA-tagged wild type (WT) ubiquitin and its lysine mutants containing only K6, K48 or K63 were used. (**C**) K48 and K63-linked ubiquitylation of endogenous STAT6 was significantly induced during KSHV reactivation. BCBL1 cells were treated with TPA and sodium butyrate (T/NB) for 24 h, followed by MG132 treatment for 8 h before harvesting. Whole cell lysates (WCLs) were lysed for denatured immunoprecipitation (IP) using antibodies specific against K48 or K63 polyubiquitin and immunoblotting (IB) as indicated in the figure.

Given that RTA can serve as an E3 ubiquitin ligase for several target substrates[30,36–38], to determine if RTA-mediated degradation of STAT6 is also dependent on its E3 ligase activity, we generated different RTA mutants within its RING finger domain (C131S, C141S, H145L, or deletion) and co-expressed them with exogenous STAT6 in 293T cells along with wild-type RTA as a parallel control. The results of the immunoblotting assay showed that site mutations (CS, CL, or CSL) in the RING domain efficiently abolished RTA-induced degradation of STAT6 (Fig 4D, right panel). Co-expressing exogenous STAT6 with different amounts of RING-deleted mutant (ΔRING) did not change the protein level of STAT6 (Fig 4D, left panel). Taken together, these results suggest that RTA promotes STAT6 degradation through its E3 ligase activity. To determine whether RTA also induces STAT6 ubiquitylation, we performed experiments in ubiquitin-modified in cells by co-expressing exogenous STAT6 with or without wild-type or RING-mutated RTA in the presence of HA-tagged ubiquitin in 293T cells, followed by treatment with the MG132 proteasome inhibitor. The results of co-IP assays with antibody against HA showed that a clear band of modified STAT6 with ubiquitin was only observed in the presence of wild-type RTA, but not in the RING-CS mutant (Fig 5A). This ubiquitin modification was confirmed by co-IP assays using FLAG (exogenous STAT6) antibody (Fig 5B, left panel), indicating that RTA promotes STAT6 ubiquitylation via its E3 ligase activity. Since both proteasome and lysosomal inhibitors impair the RTA-induced degradation of STAT6, to identify which lysine-linked form of ubiquitin participates in the modification of STAT6 induced by RTA, we performed similar experiments in ubiquitin-modified, with the exception that wild-type ubiquitin was replaced with mutants K6, K48, and K63 (all lysines within ubiquitin were mutated and only K6, K48, or K63 remained, respectively). The results showed that with the exception of K6, both K48- and K63-linked ubiquitin modification of STAT6 appeared in the presence of wild-type RTA instead of its RING-domain mutant (Fig 5B). In agreement with this finding, the K48-linked form of ubiquitinated endogenous STAT6 was also significantly increased in BCBL1 cells with reactivation of the lytic cycle, although the K63-modified form was highly latent and increased less in response to the reactivation of the lytic cycle compared with the K48-modified form (Fig 5C). These data indicate that RTA promotes K48- and K63-linked ubiquitination of STAT6.

### STAT6-mediated inhibition of TRIML2 is released by KSHV reactivation of the lytic cycle for prolonged cell survival and viral production

**Fig 6:**
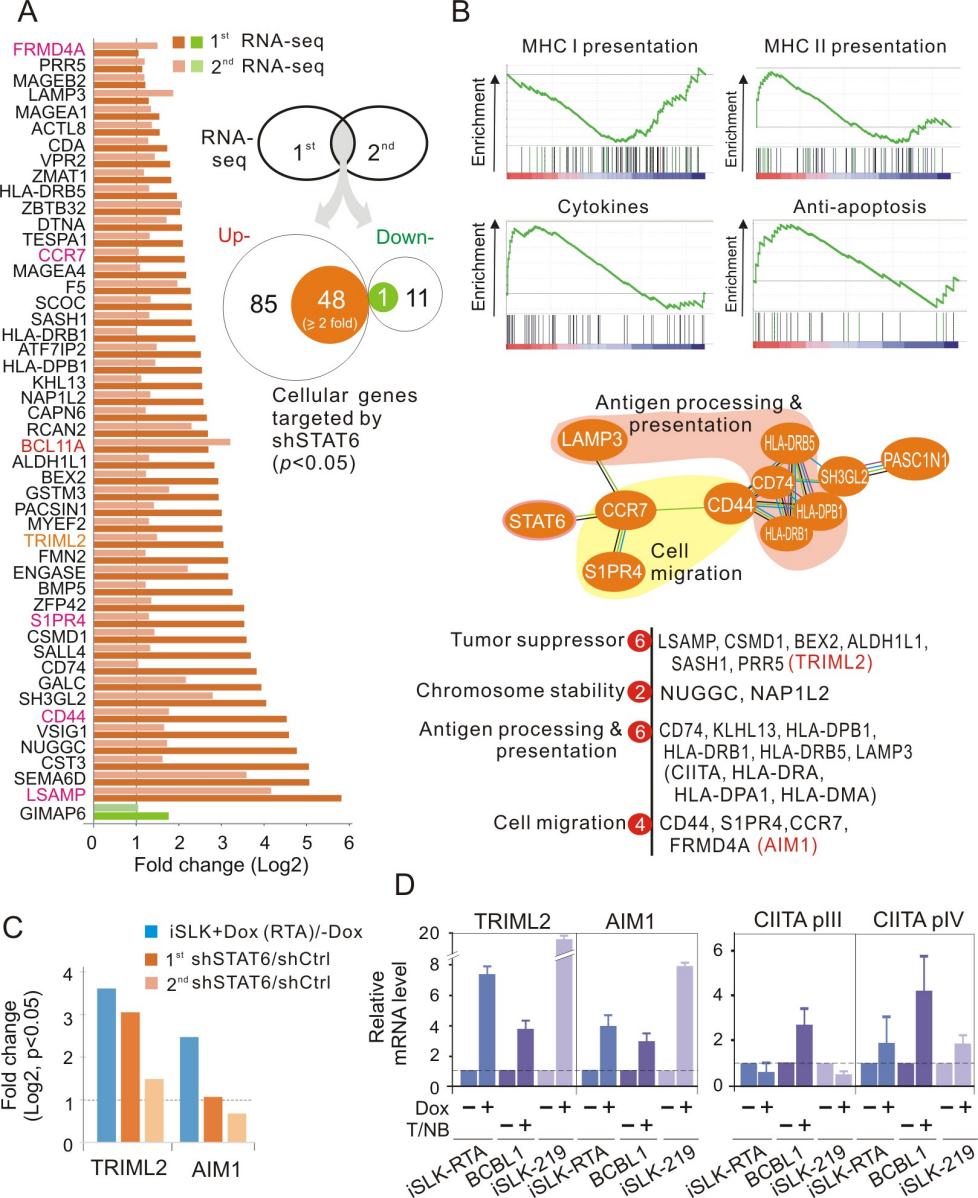
STAT6 knockdown significantly turns over cellular gene expression of PEL cells. (**A**) The expression of 49 cellular genes was significantly affected by STAT6 knockdown in PEL cells. BC3 and BCBL1 cells with or without STAT6 knockdown (S6 Fig) were individually subjected to RNA deep-sequencing analysis. The cellular genes with significantly change in the presence of STAT6 knockdown are shown. (**B**) Reduction of STAT6 led to the increased gene expression of MHC II-, cytokine- or anti-apoptosis-related proteins in PEL cells. The molecules related to antigen processing and presentation, cell migration, tumor suppressor, and chromosome stability are highlighted. (**C**) Expression of TRIML2 and AIM1 was consistently enhanced by RTA or STAT6 knockdown. The results were the RNA deep-sequencing analysis of iSLK cells with Doxycycline-inducible RTA (S5 Fig) and PEL cells with STAT6 knockdown. (**D**) Quantitative PCR analysis of TRIML2, AIM1 and CIITA expression in the iSLK-RTA and iSLK-219 cells treated with Doxycycline, or BCBL1 cells treated with TPA and sodium butyrate (T/NB) for 24 h.

To determine which cellular signaling pathways and related molecules are impaired by the RTA-induced degradation of STAT6, iSLK-RTA cells treated with Dox for 24 h to induce RTA expression, followed by transfection with exogenous STAT6 or vector alone were subjected to RNA deep-sequencing analysis. The immunoblotting assays showed that the protein level of RTA-induced degradation of endogenous STAT6 was successfully complemented with exogenous STAT6 (S5A Fig). RNA sequencing analysis showed that 563 cellular genes were significantly impaired by RTA. Interestingly, 39 genes were dramatically upregulated by transfection with exogenous STAT6, most of which encoded cellular receptors and growth factors and were mainly involved in the regulation of viral infection, metabolism, and tumor formation. In contrast, 37 genes were selectively downregulated, including those encoding cellular receptors and growth factors, particularly those involved in the mitogen-activated protein kinase/Ras signaling pathway, protein digestion, and absorption (S5B and S5C Fig), which may explain, at least in part, why STAT6 needs to be degraded by RTA during reactivation of the lytic cycle. To determine if the cellular molecules related to STAT6 degradation were cell-type independent, KSHV-infected PEL cells with stable STAT6 or scramble knockdown were individually established and subjected to RNA deep sequencing (S6 Fig). The results showed that 48 of 85 cellular genes were significantly (≥2 fold, p<0.05) upregulated in PEL cells with STAT6 knockdown, while only 1 of 11 genes was significantly downregulated (Fig 6A). Further analysis of functional pathways showed that the genes upregulated by STAT6 knockdown were the majority of cytokines, tumor suppressors, and molecules regulating MHC II-associated antigen processing and presentation, anti-apoptosis, chromosome stability, and cell migration (Fig 6B). Unexpectedly, only two genes, namely tripartite motif family like 2 (TRIML2, a tumor suppressor) and absent in melanoma 1 protein (AIM1, related to cell migration) were consistently upregulated in the two screening systems of iSLK-RTA and PEL cells with STAT6 knockdown (Fig 6C). Nucleotide sequence analysis revealed that both TRIML2 and AIM1 promoters contained STAT6-binding sites (S7A Fig), and chromatin IP assays against STAT6 confirmed that STAT6 bound to these two gene promoters through STAT6-binding sites, and the DNA-binding ability of STAT6 increased in reactivation of the lytic cycle (S7B Fig). To further verify that the transcription of those STAT6-mediated genes increased in the presence of RTA and reactivation of the lytic cycle in a cell-type independent manner, total RNAs from BCBL1 cells with or without reactivation of the lytic cycle were subjected to qPCR for specific genes. Along with iSLK-RTA and iSLK-219 cells treated or untreated for induction of RTA as parallel controls, the results showed that the transcription levels of randomly selected genes including CIITA, EPAS1, PGF and NGF, TRIML2, and AIM1, were dramatically enhanced in both RTA-induced iSLK cells and BCBL1 upon reactivation of the lytic cycle (Fig 6D, S5D Fig). In agreement with enhanced CIITA, the transcription of MHC II molecules, including HLA-DRA, DPA, and DQB, was also increased (S5D Fig).

**Fig 7:**
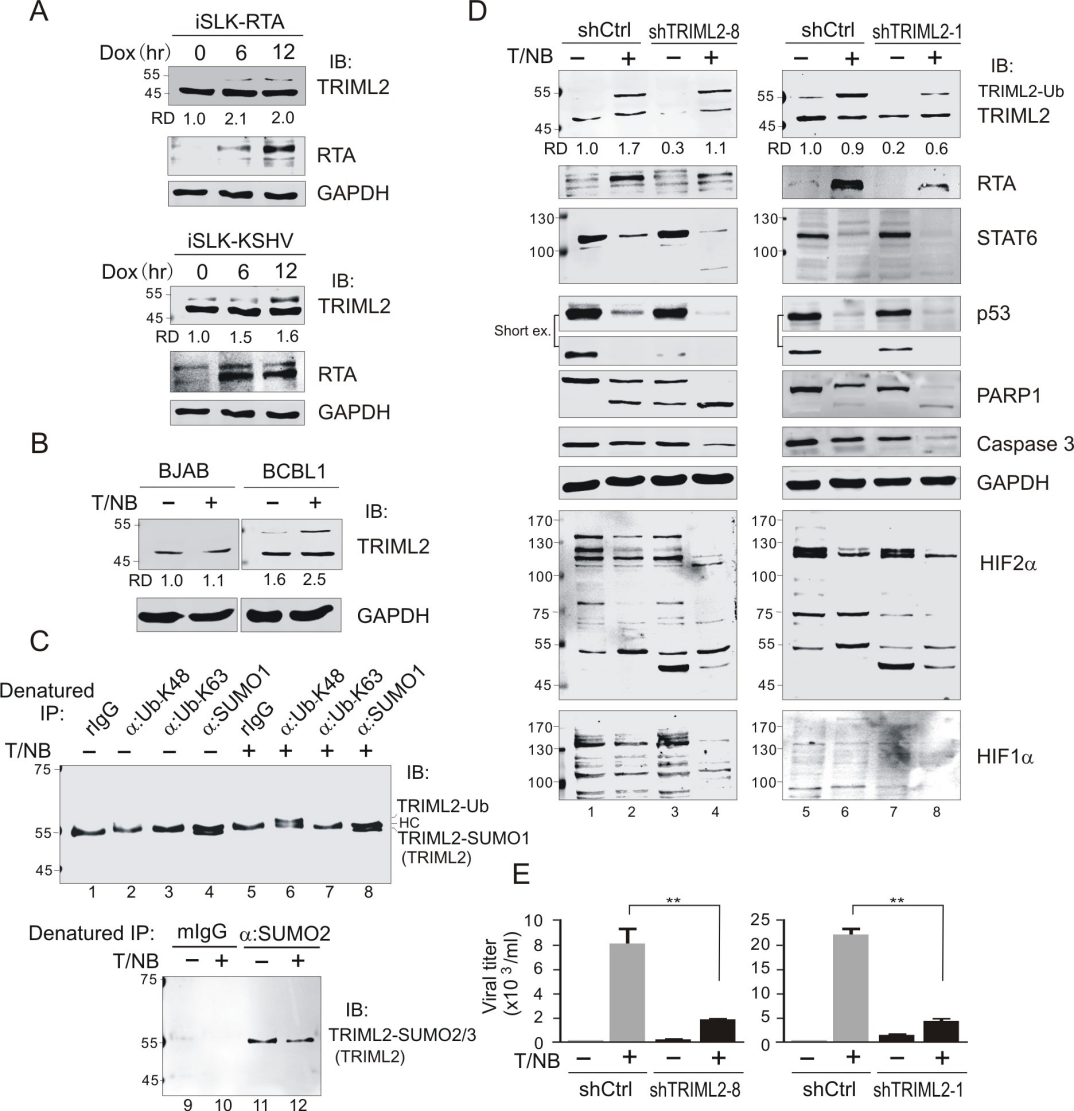
TRIML2 contributes to KSHV lytic replication and cellular anti-apoptosis. (**A**) RTA induced the expression and ubiquitylation of TRIML2. iSLK cells carrying doxycline-induced RTA (iSLK-RTA) or RTA and KSHV whole genome (iSLK-KSHV) were treated with doxycline for different time points, and subjected to immunoblotting as indicated in figure. (**B**) Reactivation of KSHV in PEL cells induced expression and ubiquitylation of TRIML2. KSHV-negative B lymphoma BJAB and KSHV-positive BCBL1 cells were individually treated with or without TPA/sodium butyrate (T/NB) for 24 h, and subjected to immunoblotting as indicated. (**C**) K48-linked ubiquitylation of TRIML2 was induced during KSHV reactivation. Whole cell lysates (WCLs) of BCBL1 described in panel B were lysed and aliquoted equally for denatured immunoprecipitation (IP) with antibodies specific against K48 or K63 polyubiquitin, SUMO1 and SUMO2/3, respectively, and immunoblotting (IB) for TRIML2. Rabbit and mouse IgG (rIgG, mIgG) were individually used as negative control. (**D**) Knockdown of TRIML2 reduced the expression of RTA and anti-apoptosis ability of KSHV-infected PEL cells. BCBL1 cells with TRIML2 (shTRIML2-8, or shTRIML2-1) or luciferase control (shCtrl) stable knockdown were individually treated with or without TPA/NaB (T/NB) for 24 h, and subjected to immunoblotting as indicated. (**E**) TRIML2 knockdown reduced TPA- and sodium butyrate-induced KSHV virion production. The supernatants from equal amounts cells in panel D were purified to quantitate virion production. The statistical significance was evaluated and *p*<0.05 indicated as double asterisks.

To further confirm that the level of TRIML2 expression increased in the presence of RTA, we examined the expression of TRIML2 in RTA-induced iSLK cells and KSHV-iSLK cells with Dox induction for different time points. Consistent with the reduction of STAT6 (Fig 1C), the results from immunoblotting assays showed that the level of TRIML2 expression was enhanced along with the increased expression of RTA in RTA-induced iSLK cells (Fig 7A, top panel). In contrast, a similar phenomenon was observed in KSHV-iSLK cells with reactivation induction, although a high migrating band (~55kDa) of modified TRIML2 was revealed (Fig 7A, bottom panel). Interestingly, a similar effect of increased TRIML2 and its modified (~55 kDa) form consistently appeared in BCBL1 cells instead of BJAB cells treated with TPA and sodium butyrate upon reactivation of the lytic cycle (Fig 7B). To determine if the modified form was TRIML2 of !55 kDa, the cell lysates of BCBL1 with or without reactivation were individually subjected to denatured co-IP by using specific antibodies against ubiquitin (K48- or K63-linked form), SUMO1, and SUMO2/3, respectively. The results showed that TRIML2 of ~55 kDa underwent both SUMO1 and SUMO2/3 modification during KSHV latency, while SUMO2/3 and not SUMO1 modification of TRIML2 moderately decreased during lytic reactivation of KSHV (Fig 7C). In contrast, TRIML2 underwent ubiquitin modification in its K48- and not K63-linked form during the lytic cycle instead of latency (Fig 7C), indicating that the higher TRIML2 band of ~55 kDa molecular weight was its ubiquitin-modified form.

To determine the role of 55 kDa TRIML2 in KSHV latency and reactivation of the lytic cycle, we knocked down the expression of TRIML2 by targeting two different sequences (816 and 17 nt) in BCBL1 cells and then treated them with or without TPA and sodium butyrate for reactivation followed by immunoblotting assays. The results showed that the protein levels of p53 and hypoxia-inducible factor 2 alpha (HIF2α, a stress response marker) were dramatically reduced, and PARP1 (an apoptosis marker) was activated for cleavage once TRIML2 was knocked down in latency, although the protein levels of RTA, STAT6, and HIF1α did not significantly change (Fig 7D, compare lane 1 with lane 3). In contrast, during reactivation, TRIML2 knockdown not only caused further degradation of p53, HIF2α, and PARP1, but also reduced the levels of HIF1α and caspase 3 (Fig 7D, compare lane 2 with lane 4), indicating that TRIML2 contributes to the cell stress response and prolonging survival. Surprisingly, although lower levels of STAT6 and RTA expression were also observed upon TRIML2 knockdown, the ubiquitin-modified form of TIRML2 induced by RTA was impaired slightly by two different targeting sequences of TRIML2 (shTRIML2-8 and shTRIML2-1). This indicates that ubiquitin-modified TRIML2 is required for reactivation of the lytic cycle and may contribute to virion production. To confirm this speculation, the quantitative detection of virion production was conducted in the supernatants of the cell cultures. The results showed that TRIML2 knockdown did result in significantly reduced (~4 fold) virion production induced by reactivation (Fig 7E). In addition, consistence with the observation that introduction of exogenous STAT6 could significantly reduce the virion release from RTA-induced iSLK-Bac16 cells (S5A Fig, bottom panel), knockdown STAT6 expression in BCBL1 cells not only leads to dramatically increased expression of TRIML2 and its ubiquitylated form, but also enhances expression of RTA and virion production (S8 Fig), supports the notion that STAT6 plays a critical role in controlling KSHV life cycle.

### STAT6 degradation and ubiquitylated TRIML2 occur in the lytic cycle of EBV, HCMV, and HSV1

**Fig 8:**
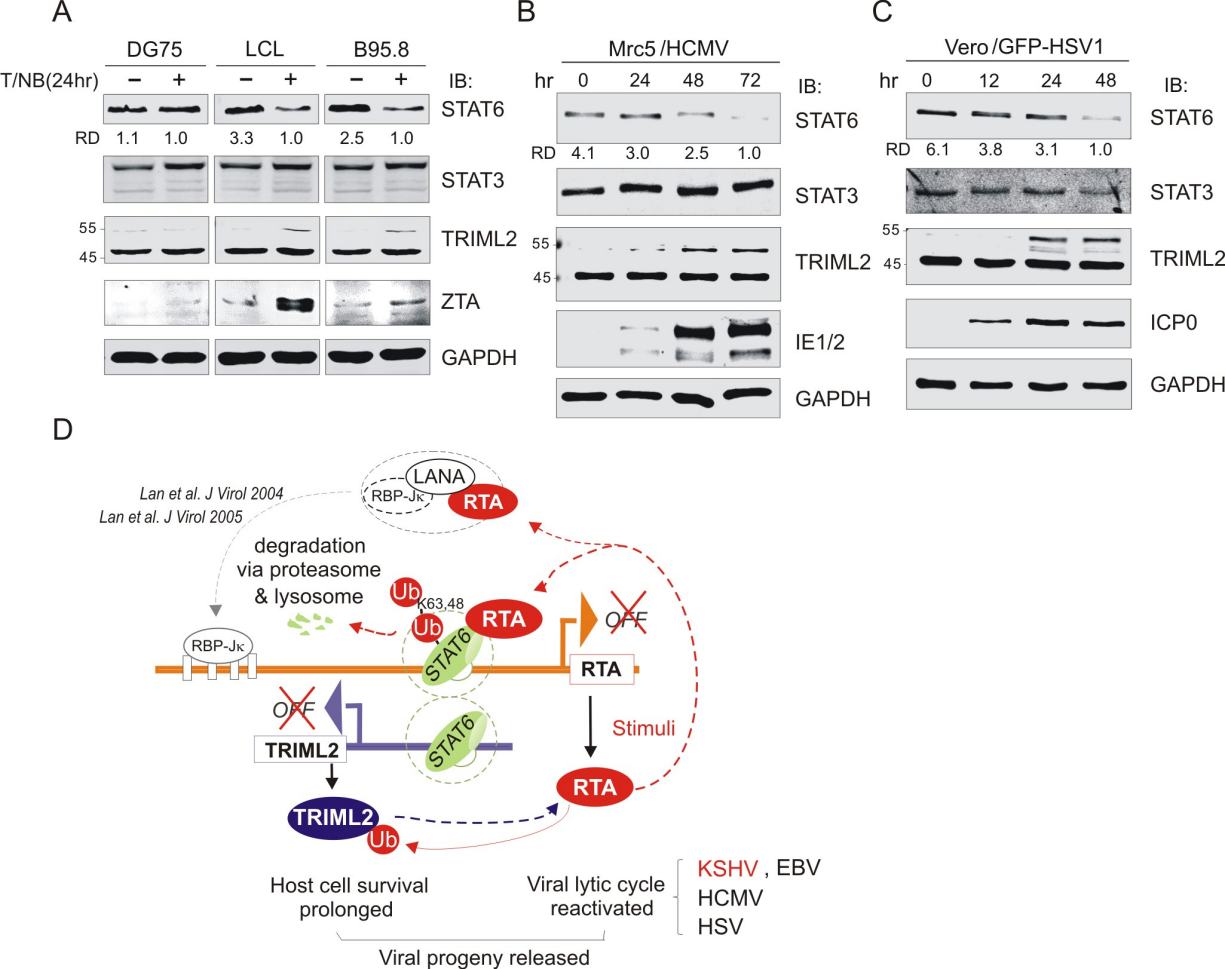
Low expression of STAT6 and high TRIML2 ubiquitylation were present during EBV, HCMV, and HSV1 lytic replication. (**A**) EBV reactivation led to inhibition of STAT6 expression and increased monoubiquinated TRIML2. Whole cell lysates from EBV-positive (LCL and B95.8) and negative (DG75) B lymphoma cells with TPA and sodium butyrate treatment for 24 h, were individually subjected to immunoblotting as indicated in the figure. (**B**) and (**C**) De novo infection of HCMV and HSV1 led to inhibition of STAT6 expression and increased monoubiquinated TRIML2 in early lytic replication. Mrc-5 (human fetal lung fibroblast cells) and Vero (monkey kidney epithelial cells) were individually infected with HCMV and HSV1 at an MOI of 3 and 0.1 as described in material and method. Cells were harvested at different time points and lysed for immunoblotting as indicated in the figure. RD, relative density. (**D**) Proposed model of RTA-induced degradation of STAT6 upon KSHV reactivation. During reactivation of KSHV latently-infected cells by stimuli like TPA and sodium butyrate, KSHV encoded RTA not only blocks formation of LANA-RBP-Jκ complex (Lan et al, J Virol 2004, 2005), but also induces K48- and K63-linked ubiquitylation of STAT6 for degradation via proteasome and lysosome. The interaction between STAT6 and RTA not only leads to STAT6 degradation but also increase TRIML2 expression and ubiquitylation, which in turn prolongs host cell survival in lytic replication and enhances RTA expression, and facilitates viral progeny production. Similar effects are observed in other human herpesviruses including EBV, HCMV and HSV1 as indicated.

To determine if STAT6 degradation and ubiquitylated TRIML2 occur during reactivation of the lytic cycle in other human herpesviruses, we also detected the protein levels of STAT6 and TRIML2 in latently EBV-infected B cells with or without reactivation, as well as in permissive cells with primary infection of HCMV or HSV1 at different time points. Interestingly, the results showed that reactivation by TPA and sodium butyrate exclusively led to STAT6 degradation and enhancement of ubiquitylated TRIML2 in LCL and B95.8 cells latently infected with EBV instead of uninfected DG75 cells (Fig 8A). In the permissive cells Mrc5 and Vero with primary infection of HCMV and HSV1, respectively, we observed that the protein level of STAT6 was gradually inhibited along with the increase expression of lytic genes (IE1/2 in HCMV, ICP0 in HSV1), while ubiquitylated TRIML2 was enhanced (Fig 8B and 8C). These data indicate that STAT6 degradation and ubiquitylated TRIML2 also occurred during reactivation of the EBV, HCMV, and HSV1 lytic cycle. Taken together, these data supports the conclusion that degradation of STAT6 and ubiquitylated TRIML2 are essential for lytic activation of herpesviruses.

## Discussion

The lytic phase of KSHV is critical for its oncogenesis and pathogenesis. Previous studies have reported that extracellular stimulation of IL-4 reactivates the KSHV lytic cycle[33], while KSHV not only blocks the IL-4-induced phosphorylation of STAT6 during latency[15], but also induces nuclear localization and cleavage of STAT6 to inhibit the transcription of RTA and blocks lytic replication[34]. In this study, we provided additional evidence for STAT6 as a key molecule that responds to lytic stimuli. Consistent with previous studies, we found that the degradation of STAT6 is induced upon KSHV reactivation. During reactivation of KSHV latently infected cells by stimuli, such as TPA and sodium butyrate, KSHV-encoded lytic antigen RTA not only blocks formation of the LANA-RBP-Jκ complex as previously reported [29,39], but also induces K48- and K63-linked ubiquitylation of STAT6 for degradation via the proteasome and lysosome. The fact that inhibition of STAT6 is sufficient to activate lytic cycle, indicates that it plays a critical role in controlling the KSHV life cycle. The interaction between STAT6 and RTA resulted in STAT6 degradation and increased the expression and ubiquitylated form of TRIML2, which in turn, prolongs host cell survival in lytic replication and enhances RTA expression to facilitate viral progeny production (Fig 8D). A similar phenomenon has also been observed in other human herpesviruses, including EBV, HCMV, and HSV1, when they undergo lytic replication, indicating that STAT6 degradation and TRIML2 ubiquitylation are required for reactivation of human herpesvirus from latency to the lytic cycle.

In agreement with the observation that RTA presents in the early stage of primary infection, we also saw the increased expression of STAT6 along with reduced RTA expression in HUVEC cells with KSHV primary infection at longer time points. This suggests that the dynamic regulation of STAT6 expression is indeed critical for the KSHV life cycle. Moreover, given previous studies showing that the LANA-binding region of STAT6 is located at its amino terminus and this interaction leads to cleavage of the TAD from STAT6 to maintain viral latency[34], it is not difficult to understand why RTA interacts with STAT6 through the TAD. KSHV consistently induces the nuclear localization of STAT6 independently of Y641 (stimulated by IL-4 or IL-13) in latency. RTA-induced degradation of STAT6 is also independent of phosphorylation on Y641 during the lytic cycle, although the Y641F mutant blocked the inhibitory role of RTA on exogenous STAT6 to some extent (which could be due to the lose ability of Y641F in shuffling between nuclear and cytoplasm).

Despite previous reports that autophagy is activated concomitantly with early induction of KSHV lytic cycle in PEL cells[40], our finding that RTA-induced STAT6 degradation is impaired by both proteasome and lysosome inhibitors suggests that the degradation of STAT6 is critical for reactivation of KSHV lytic cycle, and needs to be strictly controlled via both proteasome and lysosomal pathways. Although it has been shown that RTA targets many substrates for degradation via the proteasome pathway, this is the first report that RTA also induces target protein degradation by regulating the lysosomal pathway. The observation that punctate dots of STAT6 co-localize with LC3B in the cytoplasm in the presence of RTA expression supports our speculation. Interestingly, consistently higher levels of K48- than K63-linked ubiquitylated in exogenous and endogenous STAT6 induced by RTA were observed. The slightly slower migration of the K48-modified rather than K63-modified form could have been due to the additional modification.

Different from the Cbl-induced ubiquitylation of STAT6 on both K108 and K398[35], the fact that mutations of K108 and K398 of STAT6 did not block the ability of RTA to induce STAT6 degradation indicated that RTA-induced ubiquitylation of STAT6 is independent of K108 and K398. The results of protein levels of STAT6 truncated mutants in the presence or absence of RTA indicate that the potential ubiquitylated residues of RTA-induced STAT6 are likely located at the region of the SH2 domain. However, the specific residue of STAT6 induced by RTA for ubiquitylation requires further investigation. Considering the fact that RTA did not degrade endogenous phosphorylated STAT6 on Y641, the mutation of Y641F efficiently blocked the degradation of exogenous STAT6 induced by RTA, indicating that it could be another modification instead of phosphorylation of STAT6 on Y641 that is required for RTA-mediated STAT6 degradation in the lytic cycle. Of course, it is possible that another molecule is involved in the regulation of endogenous STAT6 phosphorylation on Y641, which blocks the ability of RTA to induce degradation of phosphorylated STAT6.

To determine which molecules and related functional pathways are impaired by RTA-mediated STAT6 degradation in a cell-type independent manner, we performed RNA deep-sequencing analysis using iSLK cells with RTA-induced expression, or PEL cells with the knockdown of STAT6. Although very few genes with significant changes in the two-screening systems overlapped, the genes, including cell surface molecules (such as MHCII, the cellular receptor), cytokines, and stress responder (such as anti-apoptosis) were consistently impaired, indicating that STAT6 controls extracellular signaling transduction, antigen presentation, and cell survival and growth, thereby facilitating virion production upon the reactivation of the lytic cycle. Sequence analysis data revealed that most promoters of STAT6-mediated genes, such as TRIML2 and AIM1 targeted by RTA contained STAT6-binding sties, potentially explained why RTA directly inhibits STAT6 expression.

TRIML2, one of the genes targeted by the RTA-mediated degradation of STAT6, is a p53-associated tumor suppressor; however, its biological function remains largely unknown. Our study showed that the expression of total and ubiquitylated TRIML2 was significantly increased by RTA during the lytic cycle. Although the knockdown of TRIML2 led to the reduced expression of p53, the activation of PARP1 and cleavage of HIF2α did not affect the protein level of STAT6 in latency. Compared with the scramble knockdown control, the reactivation of BCBL1 cells with TRIML2 knockdown by TPA and sodium butyrate significantly reduced the expression of most genes, including RTA, indicating that TRIML2 plays a critical role in prolonged cell survival. We observed a clear slow migrating band (~55 kDa) of TRIML2 in the presence of endogenous or exogenous RTA, and we found that it was the K48-linked ubiquitin modified form of TRIML2 by using the IP assay. In contrast, a slight reduction of SUMO-2/3-modified TRIML2 was observed, while no change was observed of the SUMO1-modified form or K63-linked ubiquitin modified form of TRIML2.

In accordance with previous reports that STAT6 deficiency in mice leads to higher susceptibility to viral infection [4], our study showed that the inhibition of STAT6 by small hairpin RNA interference upregulated RTA expression, which was further enhanced by RTA-mediated degradation of STAT6 for lytic replication and virion production. In contrast to STAT6, we did not see any apparent degradation of STAT3 in the reactivation of KSHV, EBV or HCMV lytic cycle, albeit a gradual inhibition of STAT3 expression occurred in the late stage of HSV1 or KSHV primary infection. In addition, the degradation of STAT6 occurred not only in the lytic cycle of KSHV, but also in the lytic cycle of EBV, and lytic stage of primary infection with HCMV or HSV1, confirming the notion that the degradation of STAT6 is specifically induced by all human herpesviruses for the reactivation of the lytic cycle. The observation of consistently increased levels of ubiquitylated TRIML2 along with STAT6 degradation in the lytic phase of all herpesviruses strongly suggest that TRIML2 is a key molecule in response to reactivation of lytic cycle. Taken together, our results reveal a previously uncharacterized pathway for KSHV and other human herpesviral pathogenesis, which includes the degradation of STAT6.

## Materials and methods

### DNA constructions

FLAG-STAT6 was a gift from Jaharul S. Haque at Lerner Research Institute. Plasmids expressing STAT6 truncation mutants (ΔN, ΔDBD, ΔTAD and Y641F) were described previously[34], STAT6 mutants K108R, K398R, 2KR (K108,398R), 2KR+Y641F were individually constructed by PCR-directed site mutation. The luciferase reporter STAT6p-luc driven by wild type STAT6 promoter containing RBP-Jκ binding sites was constructed by PCR amplicon (with BJAB genome as template) and inserted into pGL3 vector with *Mlu*I and *Bgl*II restriction enzyme sites. The RTA ΔRING (E3 ubiquitin ligase activity domain from 118 to 207 aa was deleted amino acid) with myc or RFP tag was constructed by overlap PCR and inserted into pA3M or pDsRed1-N1 vector through *Eco*RI and *Xba*I sites or *Eco*RI site, respectively. The RTA site mutants CS (C131S, C141S), CL (C131S, H145L), and CSL (C131S, C141S, H145L) were generated by PCR-site directed mutation with pA3M-RTA as template. pEGFP-LC3B expressing GFP-tagged LC3B was obtained by PCR amplicon (with BJAB genome as template) and subcloned into pEGFP-C1 vector at *Eco*RI and *Bam*HI sites. The RFP-RTA was generated by the cDNA fragment of RTA from pGEX-RTA modified plasmid (with site directed mutation of RTA stop codon TGA into AGA) digested by *Eco*RI, and inserted into pDsRed-N1 to generate pDsRed-RTA. The plasmid expressing MyD88-FLAG were generated by PCR amplicon (with BJAB genome as template) inserted into pcDNA3.1-FLAG vector with *Bam*HI and *Eco*RV digestion sites. Plasmids LANA-myc, and pCDNA3.1-GFP-NLS-myc was described previously[34]. The plasmid expressing HA-Ubiquitin with lysine 6 (K6), lysine 48 (K48) or lysine 63 (K63) only was stored in our laboratory.

### Reagents and antibodies

Antibodies to STAT6 (D3H4, Cell Signaling technology), p-STAT6 (Tyr-641, Cell Signaling Technology), FLAG (M2, Sigma), p53 (Do-1, Santa cruz), PARP1 (F2, Santa cruz), Caspase-3 (H-277, Santa cruz), TRIML2 (ARP55636-P050, Aviva System Biology), EBV ZEBRA (sc-53904, Santa cruz), ICP0 (11060, Santa cruz), Ub-K48 (05–1307, EMD Millipore),Ub-K63 (05–1308, EMD Millipore), LC3B (GT3612,GeneTex) and GAPDH (G8140-01, US Biological) were used according to the manufacturers specifications. The monoclonal antibody anti-myc (9E10) and HA (12CA5) were prepared from hybridoma cultures. Mouse monoclonal antibodies against RTA were kindly provided by Ke Lan from Shanghai Pasteur Institute of CAS. Antibodies against IE1/2 were kindly provided by Zhikang Qian from Shanghai Pasteur Institute of CAS. Tetradecanoyl Phorbol Acetate (TPA) was purchased from Sigma and sodium butyrate from J&K Corporation. Proteasome inhibitor MG132 was purchased from Biomol International. 3-Methyladenine (3-MA), Choloquine (Chl), Cyclohexamide (CHX) and Hygromycin B were purchased from Sigma–Aldrich. Doxycycline (DOX) was purchased from Sangon Biotech (Shanghai). PMSF, Leupeptin, Aprotinin, Pepstatin A and Puromycin were purchased from Amresco, and G418 from Inalco S.p.A.

### Cell culture and transfection

KSHV and EBV-negative (BJAB and DG75 from American Type Culture Collection [ATCC], Manassas, VA), KSHV-positive (BC3 and BCBL1 from ATCC) B-lymphoma cells, EBV-positive cells (B95.8 from ATCC and EBV-transformed primary B cell lines LCL *in vitro* generated in the lab[41]), iSLK (1mg/ml puromycin, 250ug/ml G418, a gift from Shou-Jiang Gao at University of South California) and iSLK-Bac16 (K-iSLK, 1mg/ml hygromycin, 250ug/ml G418 and 1ug/ml puromycin, a gift from Shou-Jiang Gao at University of South California) and iSLK.219 (400 μg/mL hygromycin, 250 μg/mL G418, and 4 μg/mL puromycin[42]) cells were maintained in DMEM medium supplemented with 10% fetal bovine serum (FBS) and 1% penicillin and streptomycin (Gibco-BRL). HUVEC (ATCC), and HEK293 (ATCC) cells, were maintained in DMEM supplemented with 10% FBS. All cell lines were incubated at 37°C in a humidified environmental incubator with 5% CO_2_. 293 cells were transfected with 1 mg/ml polyethyleneimine (PEI) at a ratio of 1μg plasmid DNA: 3μl PEI. iSLK and iSLK-Bac16 were transfected with Lipofectamine 2000 reagent (Invitrogen) according to the manufacturer’s recommendations. Cells were cultured 24hrs before transfection with cell confluence reaching 60–70%.

### Immunoprecipitation and immunoblotting

Immunoprecipitation (IP), immunoblotting (IB) and Immunofluorescence assays were performed as described previously [34]. Briefly, cells were harvested, washed once with ice-cold PBS, and lysed in ice-cold RIPA buffer with constant shaking. For denature IP, cells were lysed in ice-cold RIPA buffer with 1% sodium dodecyl sulfate (SDS) and boiled for 10 min. Cell debris was removed by centrifugation and the supernatants were then transferred to a new eppendorf tube. Five percent of the supernatant was used as input. The rest lysates were then precleared and beads were spun out, washed with ice-cold RIPA buffer for four times and re-suspended with RIPA buffer. Supernatant was then incubated with primary antibody for overnight. Protein of interest complexes were captured with protein A/G Sepharose beads and boiled in loading buffer, proteins were fractionated by SDS-PAGE, and transferred to nitrocellulose membrane. The protein of interest in the membrane was probed with primary antibodies, followed by incubation with appropriate secondary antibodies and canned with an Odyssey Infrared scanner (Li-Cor Biosciences). Densitometric analysis was performed with the Odyssey scanning software.

### Immunofluorescence assays

Transfected cells were cultured in coverslips, and cells were washed with ice-cold PBS twice, and fixed in 4% paraformaldehyde. After fixation, cells were washed three times in PBS and permeabilized in PBS containing 0.2% fish skin gelatin (G-7765; Sigma) and 0.2% Triton X-100, and cell nucleus was counterstained with 4, 6-diamidino-2-phenylindole (DAPI), followed by visualized with Leica SP8 confocal microscope.

### RNA sequencing and analysis

iSLK-RTA cells induced with or without DOX for 24hrs, iSLK-RTA cells induced with DOX and transfected with FLAG-STAT6, as well as BCBL1-Sramble and BCBL1-shSTAT6 were harvest and total RNA from each group was extracted using TRIzol regent (invitrogen) according to the manufacturer’s Instructions. cDNA library construction and the quality of cDNA library were tested with Agilent 2100, cDNA libraries were sequenced using an Illumina HiSeq2000 platform, adapter sequences, low quality sequences were removed to obtain clean reads. The remaining high-quality reads were used to map into the reference genome through TopHat2. The expression level of each gene was normalized and calculated as the value of fragments per transcript kilo-base per million fragments mapped (FPKM), which eliminates the influence of different gene lengths and sequencing discrepancies. The differentially expressed genes (DEGs) were selected with the threshold false discovery rate (FDR) < 0.05 and the absolute value of log2 Fold Change >1. The differentially expressed genes were noted with GO functional enrichment analysis and KEGG pathway analysis.

### Chromatin immunoprecipitation

Chromatin immunoprecipitation (ChIP) assay were performed as described previously [34]. Briefly, approximate thirty millions of BCBL1 cells with or without induction were cross-linked with 1.42% (vol/vol) formaldehyde. Fixed cells were scraped and washed with ice-cold PBS twice and lysed with IP buffer. Nuclear component was obtained by centrifuge and washed with IP buffer, and Chromatin was sheared with sonication to an average fragment size of 300 to 500 base pairs. Solubilized chromatin extracts were precleared by incubation with IgG antibody and centrifugation, and 5% supernatant was transferred to a new eppendorf tube to determine shearing efficiency and extract DNA as input. Rest of the sample was used for immunoprecipitation by using antibody against STAT6. Input DNA was precipitate with 2 volumes of ethanol, and washed with 70% (v/v) ethanol. The pellet was re-suspended in 100ul chelex 100 (10% w/v) and boiled for 10min and continue processing as IP sample. Purified DNA was amplified by quantitative PCR using TRIML2, AIM1 and RTA promoter specific primers as shown in S1 Table.

### Quantitative PCR

Total RNA was extracted by using TRIzol, and reverse transcribed into cDNA with TransScript First-Strand cDNA Synthesis SuperMix (Beijing TransGen Biotech Co., Ltd). The cDNA was amplified in a 20μl total volume containing 10μl SYBR green, 0.4μl each primer (10μM), 4.2ul H_2_O, and 5μl cDNA. A melting-curve analysis was performed to verify the specificities of the amplified products. The values for the relative levels of change were calculated by the threshold cycle (ΔΔCT) method, and samples were tested in triplicates. The primers used for real-time PCR were shown as in S1 Table.

### Dual-luciferase reporter assay

Transfected cells at 48hr post-transfection were lysed with 200ul Passive lysis buffer (Promega), followed by dual luciferase reporter assay according to the manufacturer’s instruction as described previously. Renilla luciferase was used as a control to normalize the transfection efficiency. Relative luciferase activity [RLU] was expressed as fold changes relative to the reporter construct alone. Assays were performed in triplicate.

### RNA interference

The DNA oligo of short hairpin RNA sequence (shRNA) against TRIML2-816 (816nt, 5’-TCCAATGTTAAATGTCTCTGG-3’), TRIML2-17 (17nt, 5’-GCCCTCAGTTACAGCACAA-3’), STAT6 (5’-GGGAGAAGA TGTGTGAAACTCTGAA-3’) and non-specific control sequence (5’-TGCGTTG CTAGTACCAAC-3’) were individually inserted into the pGIPz vector according to the Clonetech manufacturer’s instructions. The pGIPz vector was cotransfected with lentivirus packaging plasmids into HEK293T cells for 48hrs to generate virus, which was enriched by Ultracentrifugation. The packaged viruses were used to transduce BCBL1 cells and selected using 2g/ml of puromycin. The RNA interference efficiency was assessed by immunoblot analysis with TRIML2 antibody.

### De novo infection of KSHV, HCMV and HSV1 Virion

For KSHV, well grown iSLK-Bac16 cells harboring KSHV genome were induced with Induction medium (DMEM + 10% FBS + 1% Pen/Strep (Optional) + 1mM sodium butyrate + 1μg/ml doxycycline, no Hygromycin, no Puromycin, no G418) for 2days followed by incubation with maintain medium (DMEM + 10% FBS + 1% Pen/Strep + 1μg/ml Puromycin+250μg/ml G418+1.2mg/ml Hygromycin) for another 2-3days. After induction cell medium was collected, cells in medium were removed by centrifuge at 3000g for 10min. The supernatant of culture medium was filtered through a 0.45μm filter, and viral particles were spun down at 25,000 rpm for 2h at 4°C. The concentrated virus was harvested by re-suspended in RPMI 1640 medium and stored at -80°C. KSHV *de novo* infection of HUVECs was performed through spin infection. Confluent HUVECs were infected with purified KSHV viral in RPMI 1640 containing 3% FBS, in 6-well plates at an MOI of 20. Culture Plates were centrifuged at 2000 *g* for 90 min at room temperature to promote infection. After centrifugation cells were returned into incubator and continue incubate for another 4h, after incubation cells were washed with PBS for once before fresh medium was added. Infection efficiency was determined by counting the percentage of GFP-positive cells after infection for 24hrs.

For HCMV, pAD-GFP carrying the green fluorescent protein (GFP)-tagged genome of the HCMV AD169 strain was used to produce viruses. Nearly 80% to 90% confluent primary human foreskin fibroblasts (HFFs) were infected with HCMV at an MOI of 0.01. Collect culture medium as viral stock at 12 to 14 days post-infection while around 70% cells were died. Clear the cell debris by centrifuging at 4°C with 4000rpm for 20 minutes. Aliquot and freeze media as viral stock for -80°C storage. Thaw and sonicate the virus stock on ice and titer the viruses by TCID50 assay. For analysis of proteins, human fetal lung fibroblast cells (MRC-5 cells) were infected with HCMV at an MOI of 3. Cells were then harvested, washed, and lysed in the SDS sample buffer at indicated time.

For HSV-1(YK604, kindly provided by Yasushi Kawaguchi from the university of Tokyo) production, nearly 80% confluent Vero cells were infected with HSV-1 for 1h at an MOI of 0.01 and continuously incubated until complete Cytopathic effect (CPE). The infected cells were subjected to freezing and thawing for 2 or 3 times, followed by centrifuge at 2,000rpm for 5 min to remove the cell debris. The supernatant was filtered through a 0.45μm filter and stored at -80°C. The virus titer was determined by 50% tissue culture infectious dose (TCID50) assay. For HSV-1 *de novo* infection, the confluent Vero cells were infection HSV-1 at an MOI of 0.1 for 1 h. After infection, the monolayer was washed with PBS, and fresh medium was added. Infected cells were harvested at indicated time.

### Purification and quantitation of KSHV virion

The BCBL1 cells with or without TRIML2 knockdown cells were induced with 20 ng/ml of TPA and 1.5mM sodium butyrate (NaB) for 2 days at 37°C with 5% CO_2_. After induction, the supernatant of culture medium was collected and filtered through a 0.45μm filter, and viral particles were spun down at 25,000 rpm for 2h at 4˚C. The concentrated virus was collected and used for virion quantitation by quantitative PCR. Primers were showed in S1 Table. iSLK cells were treated with 2μg/ml DOX to induce RTA expression, BCBL1 and BC3 cells were individually treated with TPA and NaB for 24 hr to reactivate KSHV lytic life cycle.

## Supporting information

S1 Table Primers used in this study. (DOC)

S1 Fig RTA inhibits STAT6 expression. (**A**) RTA reduces exogenous STAT6 expression in a dose-dependent manner. HEK293T cells co-transfected FLAG-STAT6 with different dosages (0, 1, 2, 5μg) of RTA-myc were subjected to immunoblotting as indicated in the figure. GFP-NLS-myc was used as a parallel control. The relative density (RD) of exogenous STAT6 and GFP-NLS in the presence and absence of RTA is quantified based on triplicate experiments and shown at the bottom panel. (**B**) RTA but not LANA reduces exogenous STAT6 expression. HEK293T cells were co-transfected with the indicated expressing plasmids. At 24h post-transfection, cells were individually treated with or without TPA/Sodium butyrate for 24h before harvesting and lysing for immunoblotting.
(TIF)

S2 Fig RTA induces Myd88-FLAG for proteasome-mediated degradation. HEK293 cells were co-transfected with the indicated expressing plasmids. At 48hr post-transfection, cells were individually treated with or without 20μM MG132 for 3h before harvesting and lysing for immunoblotting.
(TIF)

S3 Fig Exogenous LC3 responds to different cell stress. HEK293T cells were transfected with GFP-LC3 expressing plasmid. At 24h post-transfection, cells were untreated (Mock), or individually treated with hypoxia (0.2% oxygen), TPA and sodium butyrate (TPA/NaB), and sera starvation for 12 h before fixed and nuclear staining (Blue) for immunofluorescent assays. The punctate dots of activated LC3 are indicated by arrows.
(TIF)

S4 Fig RTA did not localize with STAT6 Y641F mutant. 293T cells transfected with FLAG-STAT6 Y641F in the presence of RFP-RTA or RFP vector were subjected to immunofluorescent assays with RFP (red) and FLAG (green) antibody. Nuclei were stained with DAPI.
(TIF)

S5 Fig RTA-induced STAT6 degradation significantly turns over cellular gene expression of iSLK cells. (**A**) The iSLK cells with doyxycline (Dox)-induced RTA were transfected with exogenous STAT6 or vector alone. At 24hr post-transfection, cells were treated with doyxycline for 24hr before harvesting and lysing for immunoblotting. The relative levels of virion production in supernatant of iSLK-Bac16 with similar treatment are shown at the bottom panel. (**B**) Expressions of 76 out of 563 cellular genes significantly affected by RTA in iSLK cells were reversed by exogenous STAT6. The cells from panel A were individually subjected to RNA deep-sequencing analysis. The heat map of 76 genes was shown on the top panel. (**C**) Functional cluster analysis of RTA-regulated cellular genes blocked by exogenous STAT6. Partial functional pathways were highlighted at the bottom panel. (**D**) Quantitative PCR analysis of EPAS1, PGF, NGF and MHC II expression in the iSLK-RTA or iSLK-219 cells treated with Doxycycline, or BCBL1 cells treated with TPA and sodium butyrate (T/NB) for 24 hour.
(TIF)

S6 Fig Establishment of PEL cells with STAT6 knockdown. BC3 and BCBL1 cells were individually infected with lentivirus carrying shSTAT6 or shCtrl control. Immunoblotting analysis of endogenous STAT6 and GAPDH were carried out as indicated in the figure.
(TIF)

S7 Fig (**A**) Schematic of putative STAT6, RBP-Jκ and HIFα-binding sites within TRIML2 and AIM1 promoters. (**B**) STAT6 bound to TRIML2 and AIM1 promoter and enhanced by reactivation of lytic cycle. BCBL1 cells with or without TPA and sodium butyrate (NaB) treatment were subjected to Chromatin immunoprecipitation (ChIP) with endogenous STAT6. Non-specific rabbit IgG were used as control. The relative levels of STAT6 bound to TRIML2 and AIM1 promoter were detected by quantitative PCR, respectively. Data is presented as means±SD of three independent experiments.
(TIF)

S8 Fig STAT6 knockdown enhances the levels of RTA and TRIML2 expression and virion production in PEL cells. BCBL1 cells were individually infected with lentivirus carrying shSTAT6 or shCtrl control. Equal amounts of knockdown cells were subjected to immunoblotting analysis with antibodies against STAT6, TRIML2 and RTA, and the virion titer in the supernatant of culture media was carried out by quantitative PCR (bottom panel).
(TIF)
