# Correction: STAT6 degradation and ubiquitylated TRIML2 are essential for activation of human oncogenic herpesvirus

**DOI:** 10.1371/journal.ppat.1010579

**Published:** 2022-06-01

**Authors:** Feng Gu, Chong Wang, Fang Wei, Yuyan Wang, Qing Zhu, Ling Ding, Wenjia Xu, Caixia Zhu, Cankun Cai, Zhikang Qian, Zhenghong Yuan, Erle Robertson, Qiliang Cai

The Fig 5B K6-STAT6-Ub (HA) panel is incorrect; instead of the correct K6 panel, an image of the WT results at a shorter exposure time was used inadvertently. Fig 5 has been updated below to show the correct K6 panel. The underlying image data for the blots presented in Fig 5 are provided in the S1 File below.

The Fig 7A iSLK-KSHV GAPDH panel is incorrect, and Fig 7 has been updated to present the correct iSLK-KSHV GAPDH panel. The underlying image data for the blots presented in Fig 7 and the underlying individual level data presented in Fig 7E are provided in the S1 and S2 Files below.

In addition to the errors above, the BC3 STAT6, BCBL1 STAT3, and BC3 GAPDH panels of Fig 1A have not been prepared in line with best practice guidelines for image preparation. These panels have been replaced in the updated Fig 1 below to represent the obtained results more accurately. The underlying image data for the blots presented in Fig 1 are provided in the S1 File below. The individual level data underlying the results presented in Fig 1D, 1E, 1F and 1G are provided in the S3–S6 Files below respectively.

Furthermore, the authors would like to clarify that the similarities between the Fig 1A BCBL1 STAT6 panel and the Fig 4B BCBL1 STAT6 panel, as well as the similarities between the Fig 1A BCBL1 GAPDH panel, Fig 4B BCBL1 GAPDH panel, and the Fig 7B BCBL1 GAPDH panel are due to these panels being used to represent results obtained from the same experiments. The underlying image data underlying the results presented in Figs 1, 4 and 3 are provided in the S1 File below.

**Fig 1:**
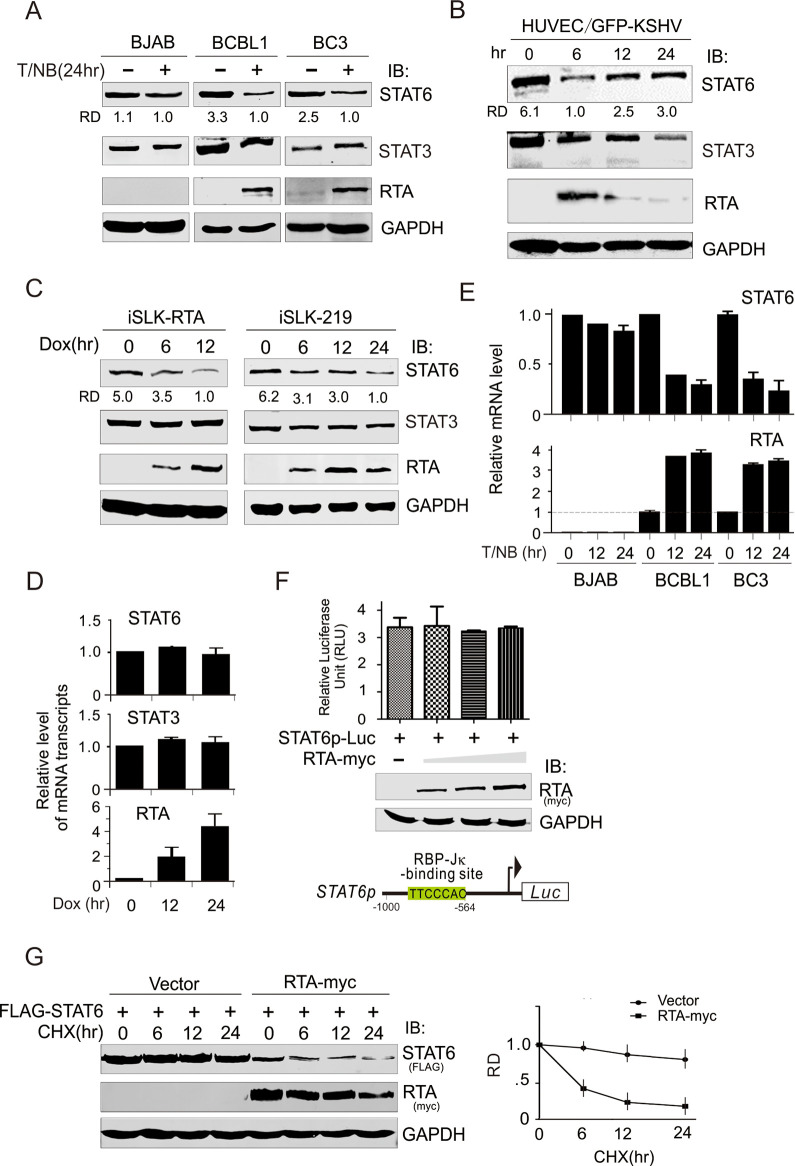
Expression of STAT6 is reduced during KSHV reactivation and early infection. (A) Expression of STAT6 but not STAT3 was dramatically reduced during KSHV reactivation. Whole cell lysates from KSHV-infected BCBL1 and BC3 and uninfected BJAB cells individually treated with or without 20 ng/ml of TPA and 1.5mM sodium butyrate (T/NB) for 24 h, were subjected to immunoblotting (IB) with antibodies as indicated in the figure. The relative density (RD) of STAT6 protein band was quantitated. (B) Early infection of KSHV reduced STAT6 expression. Whole cell lysate from HUVEC cells with KSHV infection (GFP positive) at different time points, were subjected to immunoblotting (IB) with antibodies as indicated in the figure. (C) Doxycycloline (Dox)-induced RTA expression in iSLK-RTA or iSLK-219 cells led to the decreased expression of STAT6 but not of STAT3. Whole cell lysates from the different-induction time points were subjected to immunoblotting (IB) with antibodies as indicated in the figure. (D) Quantitative PCR analysis of transcriptional level of STAT6 and STAT3 in iSLK-RTA cells with doxycline induction. (E) Quantitative PCR analysis of STAT6 and RTA mRNA transcripts in BJAB, BCBL1 and BC3 cells treated with TPA and sodium butyrate (T/NB) for 0, 12 and 24 h. The relative level of mRNA transcript was present. Beta actin was used as internal control. (F) Reporter assays of STAT6 promoter. HEK293 cells co-transfected STAT6 promoter-driven luciferase reporter with different dosage RTA (0, 1, 5, 10μg) were subjected to reporter assay. Relative firefly luciferase unit (RLU) normalization with Renilla activity was analyzed. Data is presented as means±SD of three independent experiments. The expression of exogenous RTA was verified by immunoblotting assays and shown in the middle panel. Schematic of putative RBP-Jκ-binding sites within STAT6 promoter is shown at the bottom panel. (G) RTA reduces the protein stability of STAT6. HEK293T cells were co-transfected by FLAG-STAT6 with RTA-myc or vector alone. At 36 h post-transfection, cells were treated with Cycloheximide (CHX) 200μg/ml for the indicated time before harvesting and lysing for immunoblotting. The relative density (RD) of protein level of STAT6 is quantified based on triplicate experiments and shown at the bottom panel.

**Fig 5:**
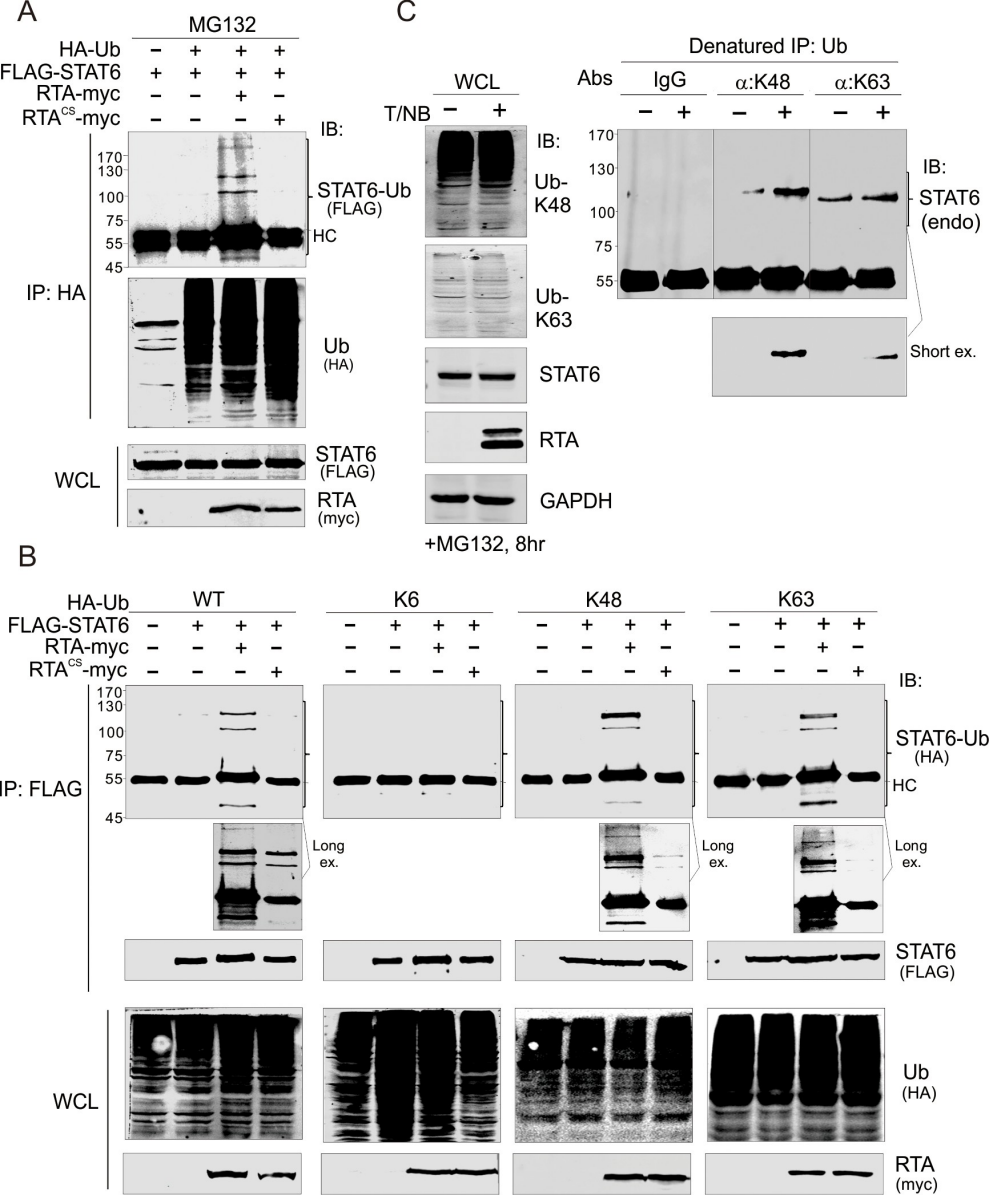
RTA promotes K48 and K63-linked ubiquitylation of STAT6. (A) RTA induced STAT6 ubiquitylation. HEK293T cells were co-transfected with different expressing plasmids as indicated. At 36 h post-transfection, cells were treated with proteasomal inhibitor MG132 for 6 h before harvesting and lysing for immunoprecipitation (IP) and immunoblotting (IB). (B) K48 and K63-linked ubiquitylation of exogenous STAT6 was induced by RTA. HEK293T cells were co-transfected and treated as in panel A. HA-tagged wild type (WT) ubiquitin and its lysine mutants containing only K6, K48 or K63 were used. (C) K48 and K63-linked ubiquitylation of endogenous STAT6 was significantly induced during KSHV reactivation. BCBL1 cells were treated with TPA and sodium butyrate (T/NB) for 24 h, followed by MG132 treatment for 8 h before harvesting. Whole cell lysates (WCLs) were lysed for denatured immunoprecipitation (IP) using antibodies specific against K48 or K63 polyubiquitin and immunoblotting (IB) as indicated in the figure.

**Fig 7:**
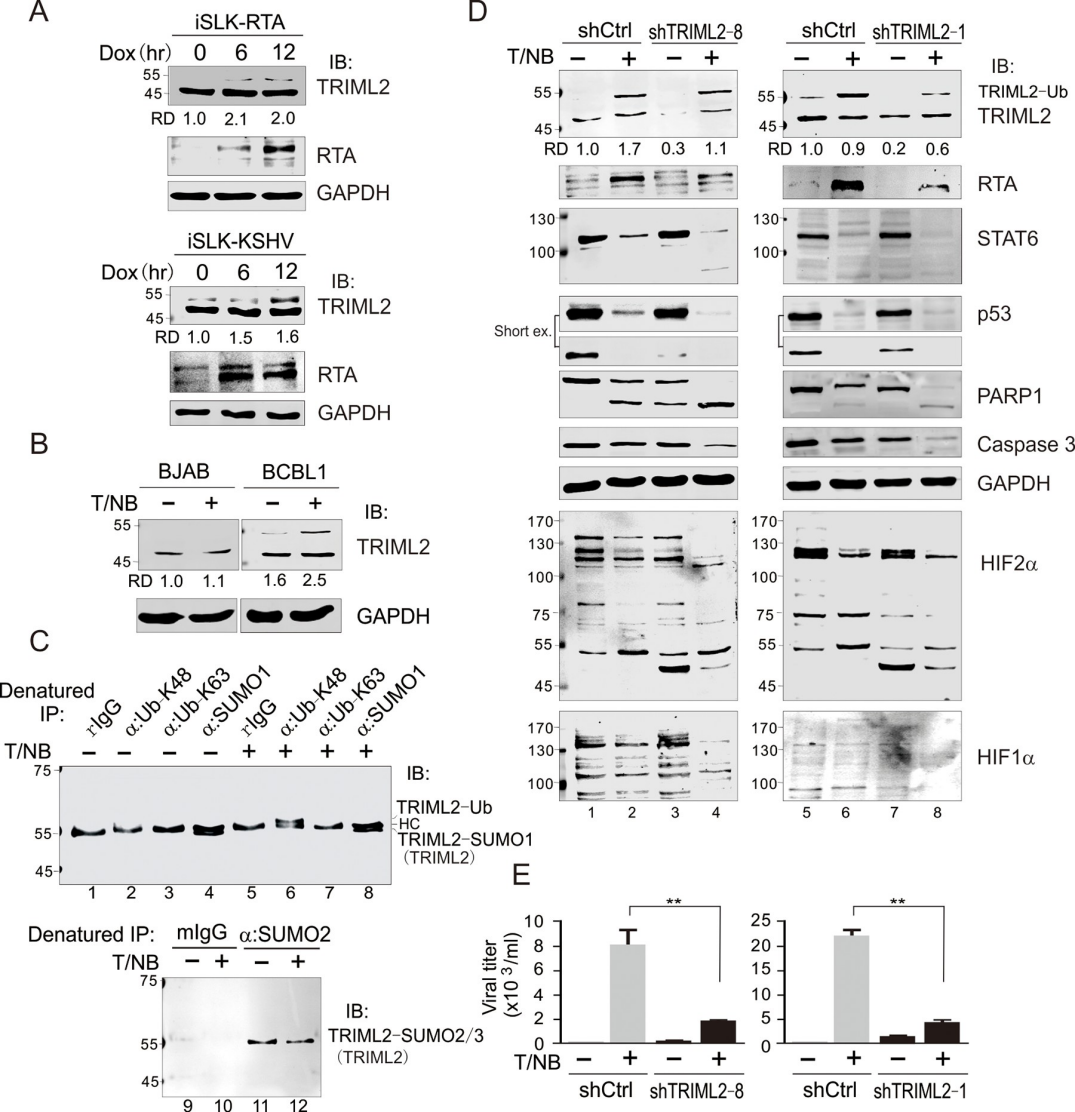
TRIML2 contributes to KSHV lytic replication and cellular anti-apoptosis. (A) RTA induced the expression and ubiquitylation of TRIML2. iSLK cells carrying doxycline-induced RTA (iSLK-RTA) or RTA and KSHV whole genome (iSLK-KSHV) were treated with doxycline for different time points, and subjected to immunoblotting as indicated in figure. (B) Reactivation of KSHV in PEL cells induced expression and ubiquitylation of TRIML2. KSHV-negative B lymphoma BJAB and KSHV-positive BCBL1 cells were individually treated with or without TPA/sodium butyrate (T/NB) for 24 h, and subjected to immunoblotting as indicated. (C) K48-linked ubiquitylation of TRIML2 was induced during KSHV reactivation. Whole cell lysates (WCLs) of BCBL1 described in panel B were lysed and aliquoted equally for denatured immunoprecipitation (IP) with antibodies specific against K48 or K63 polyubiquitin, SUMO1 and SUMO2/3, respectively, and immunoblotting (IB) for TRIML2. Rabbit and mouse IgG (rIgG, mIgG) were individually used as negative control. (D) Knockdown of TRIML2 reduced the expression of RTA and anti-apoptosis ability of KSHV-infected PEL cells. BCBL1 cells with TRIML2 (shTRIML2-8, or shTRIML2-1) or luciferase control (shCtrl) stable knockdown were individually treated with or without TPA/NaB (T/NB) for 24 h, and subjected to immunoblotting as indicated. (E) TRIML2 knockdown reduced TPA- and sodium butyrate-induced KSHV virion production. The supernatants from equal amounts cells in panel D were purified to quantitate virion production. The statistical significance was evaluated and *p*<0.05 indicated as double asterisks.

## Supporting information

S1 File Raw image data underlying Figs 1, 4, 5, and 7. (PDF)

S2 File Individual level data underlying results presented in Fig 7E. (XLSX)

S3 File Individual level data underlying results presented in Fig 1D. (XLSX)

S4 File Individual level data underlying results presented in Fig 1E. (XLSX)

S5 File Individual level data underlying results presented in Fig 1F. (XLSX)

S6 File Individual level data underlying results presented in Fig 1G. (XLSX)
