# Supplementary material for: STAT6 degradation and ubiquitylated TRIML2 are essential for activation of human oncogenic herpesvirus
Source: PLoS Pathog. 2018 Dec 10;14(12):e1007416. doi: 10.1371/journal.ppat.1007416 (PMC6287816; doi:10.1371/journal.ppat.1007416)
Supplement: S1 Table — (DOC) [file ppat.1007416.s001.doc]

**Table S1. Primers used in this study**

| Primer | sequence | Product size(bp) |
| --- | --- | --- |
| STAT6 | F: 5’-GTCTGGTCTCCAAGATGCCC-3 | 249 |
|  | R: 5’-ATATGCTCTCAAGGGTGCTGA-3’ |  |
| CIITA pIII | F: 5’-CCTGGCTCCACGCCCTG-3’ | 226 |
|  | R: 5’-GAACTGGTCGCAGTTGATG-3’ |  |
| CIITA pIV | F: 5’-GAGCTGGCGGCAGGGAG-3’ | 238 |
|  | R: 5’-GAACTGGTCGCAGTTGATG-3’ |  |
| TIRML2 | F: 5’-TGTTCAGAGTACTCCAGAGACATT-3’ | 214 |
|  | R: 5’-GTTGCCTTTTCCACGTCCAC-3’ |  |
| AIM1 | F: 5’-GAAGACTGCTGCCTGACGAT-3’ | 173 |
|  | R: 5’-TCATACTGTGTGCCCCCTTT-3’ |  |
| Beta-actin | F: 5’-GGCATCCTCACCCTGAAGTA-3’ | 82 |
|  | R: 5’-AGGTGTGGTGCCAGATTTTC-3’ |  |
| KSHV ORF72 | F: 5’- GTTCCACTGCCGCCTGTA -3’ | 609 |
|  | R: 5’- TATTTGGGACCTTTCAACAATCTCTT -3’ |  |
| ChIP-TRIML2p 1 | F: 5’-GTTTTCGAGCTAAGTGGCTTT-3’ | 102 |
|  | R: 5’-GACGTTGTGCCCATCTTTGG-3’ |  |
| ChIP-TRIML2p 2 | F: 5’-GCATGTTGGTGACTGAACCG-3’ | 72 |
|  | R: 5’-CCTCGCTCCTTCCTCTTTGAC-3’ |  |
| ChIP-TRIML2p 3 | F: 5’-TTCGCACTCTGTTACCTCACT-3’ | 107 |
|  | R: 5’-AGGAACAGCACTCACTTACGAG-3’ |  |
| ChIP-AIM1p | F: 5’- ACCGTGAAAGAGGATCTTGTACT -3’ | 156 |
|  | R: 5’-TCTTAACTTCACAGTCGTGGC -3’ |  |
