# Supplementary figures and images for: STAT6 degradation and ubiquitylated TRIML2 are essential for activation of human oncogenic herpesvirus
Source: PLoS Pathog. 2018 Dec 10;14(12):e1007416. doi: 10.1371/journal.ppat.1007416 (PMC6287816; doi:10.1371/journal.ppat.1007416)

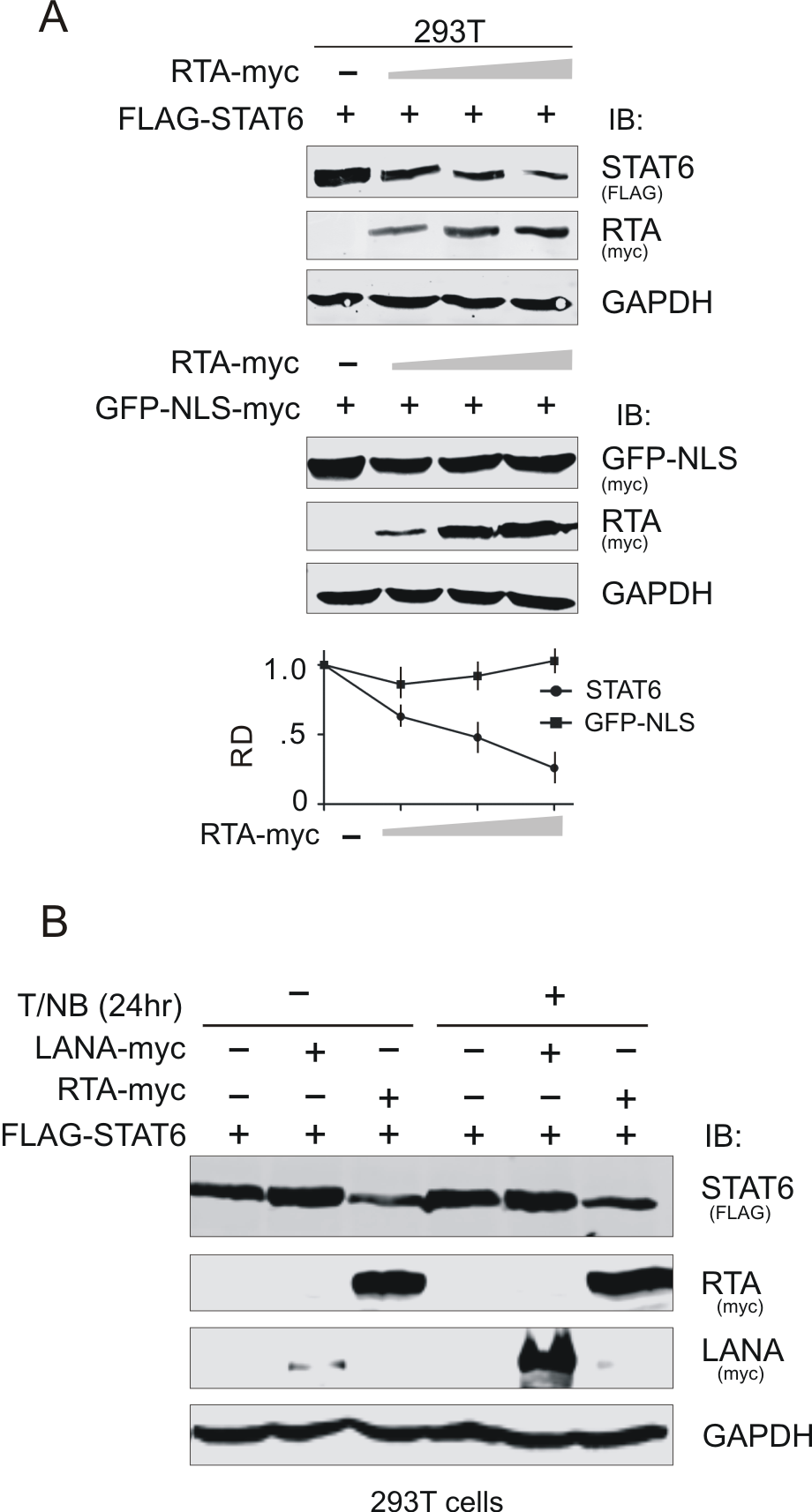

Supplement: S1 Fig — (A) RTA reduces exogenous STAT6 expression in a dose-dependent manner. HEK293T cells co-transfected FLAG-STAT6 with different dosages (0, 1, 2, 5μg) of RTA-myc were subjected to immunoblotting as indicated in the figure. GFP-NLS-myc was used as a parallel control. The relative density (RD) of exogenous STAT6 and GFP-NLS in the presence and absence of RTA is quantified based on triplicate experiments and shown at the bottom panel. (B) RTA but not LANA reduces exogenous STAT6 expression. HEK293T cells were co-transfected with the indicated expressing plasmids. At 24h post-transfection, cells were individually treated with or without TPA/Sodium butyrate for 24h before harvesting and lysing for immunoblotting. (TIF) [file ppat.1007416.s002.tif]

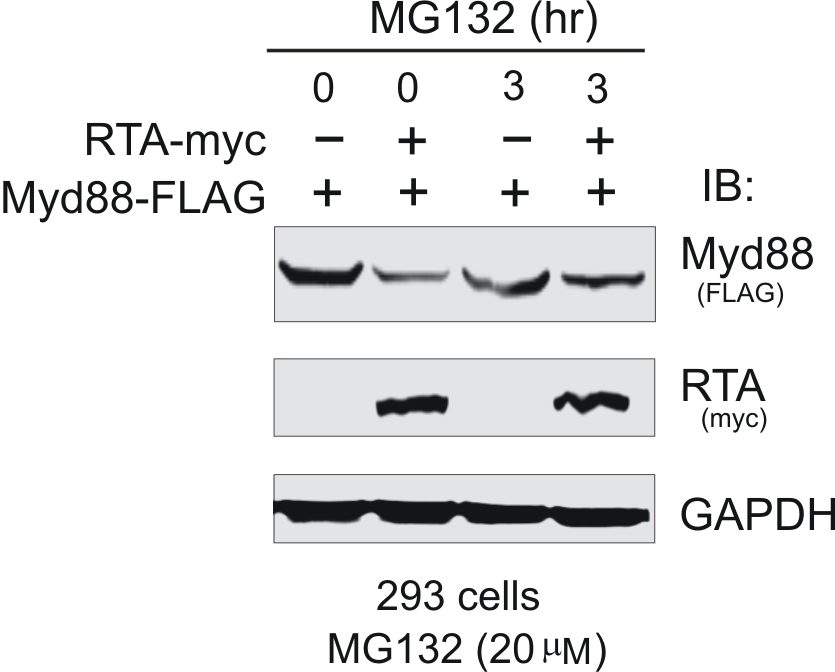

Supplement: S2 Fig — HEK293 cells were co-transfected with the indicated expressing plasmids. At 48hr post-transfection, cells were individually treated with or without 20μM MG132 for 3h before harvesting and lysing for immunoblotting. (TIF) [file ppat.1007416.s003.tif]

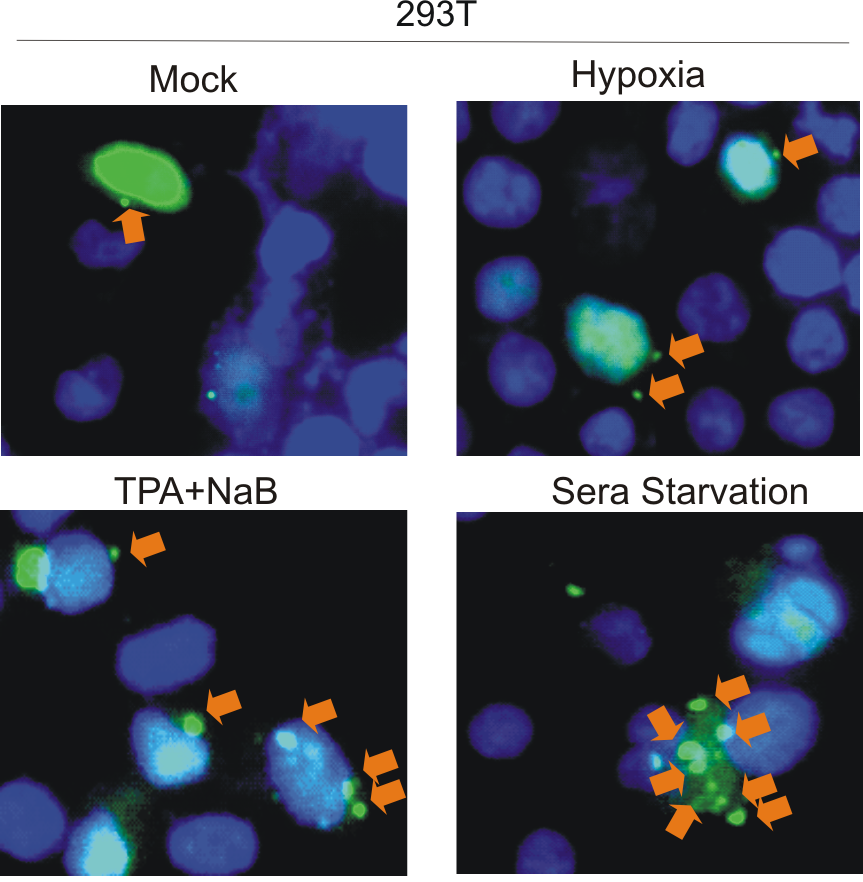

Supplement: S3 Fig — HEK293T cells were transfected with GFP-LC3 expressing plasmid. At 24h post-transfection, cells were untreated (Mock), or individually treated with hypoxia (0.2% oxygen), TPA and sodium butyrate (TPA/NaB), and sera starvation for 12 h before fixed and nuclear staining (Blue) for immunofluorescent assays. The punctate dots of activated LC3 are indicated by arrows. (TIF) [file ppat.1007416.s004.tif]

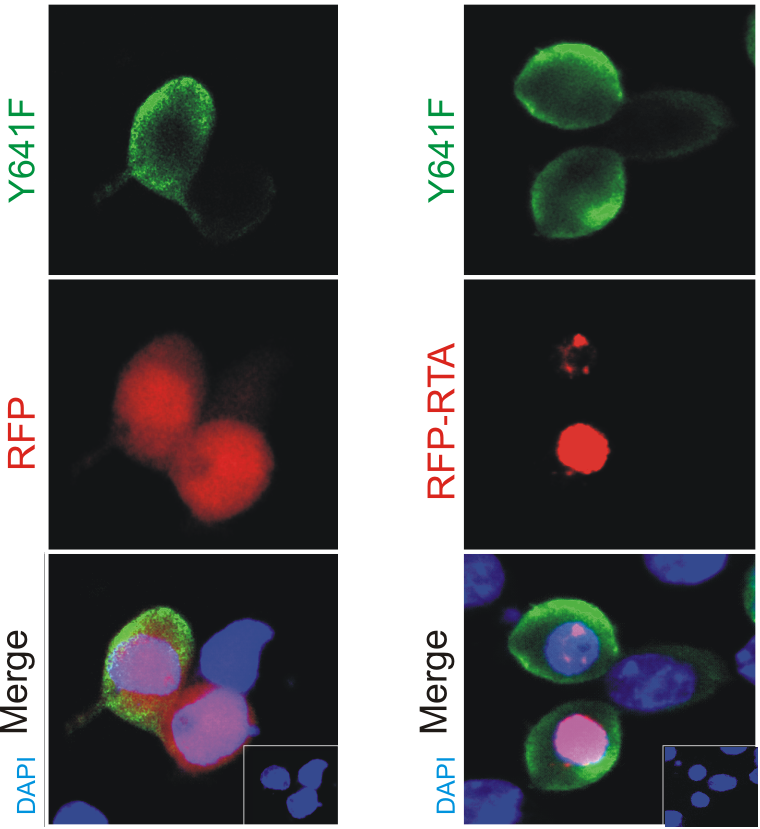

Supplement: S4 Fig — 293T cells transfected with FLAG-STAT6 Y641F in the presence of RFP-RTA or RFP vector were subjected to immunofluorescent assays with RFP (red) and FLAG (green) antibody. Nuclei were stained with DAPI. (TIF) [file ppat.1007416.s005.tif]

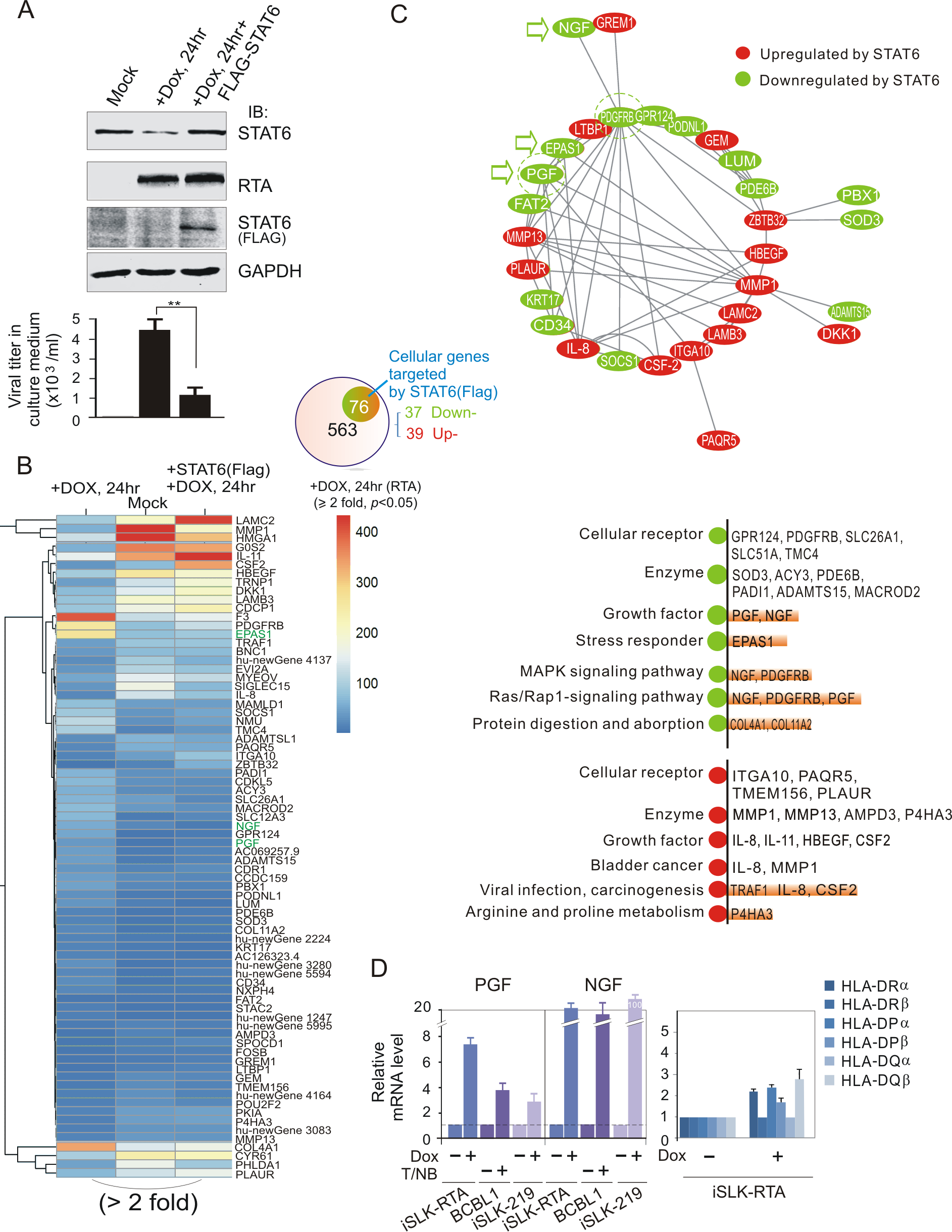

Supplement: S5 Fig — (A) The iSLK cells with doyxycline (Dox)-induced RTA were transfected with exogenous STAT6 or vector alone. At 24hr post-transfection, cells were treated with doyxycline for 24hr before harvesting and lysing for immunoblotting. The relative levels of virion production in supernatant of iSLK-Bac16 with similar treatment are shown at the bottom panel. (B) Expressions of 76 out of 563 cellular genes significantly affected by RTA in iSLK cells were reversed by exogenous STAT6. The cells from panel A were individually subjected to RNA deep-sequencing analysis. The heat map of 76 genes was shown on the top panel. (C) Functional cluster analysis of RTA-regulated cellular genes blocked by exogenous STAT6. Partial functional pathways were highlighted at the bottom panel. (D) Quantitative PCR analysis of EPAS1, PGF, NGF and MHC II expression in the iSLK-RTA or iSLK-219 cells treated with Doxycycline, or BCBL1 cells treated with TPA and sodium butyrate (T/NB) for 24 hour. (TIF) [file ppat.1007416.s006.tif]

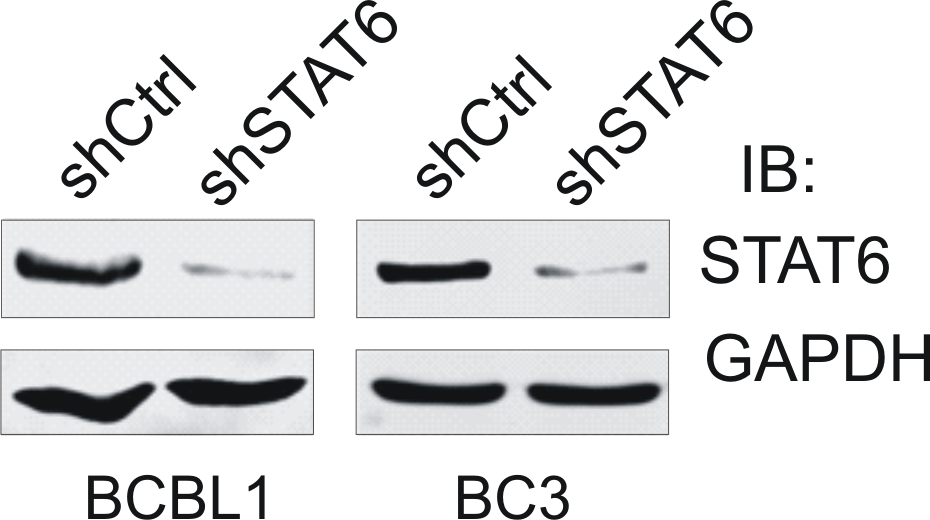

Supplement: S6 Fig — BC3 and BCBL1 cells were individually infected with lentivirus carrying shSTAT6 or shCtrl control. Immunoblotting analysis of endogenous STAT6 and GAPDH were carried out as indicated in the figure. (TIF) [file ppat.1007416.s007.tif]

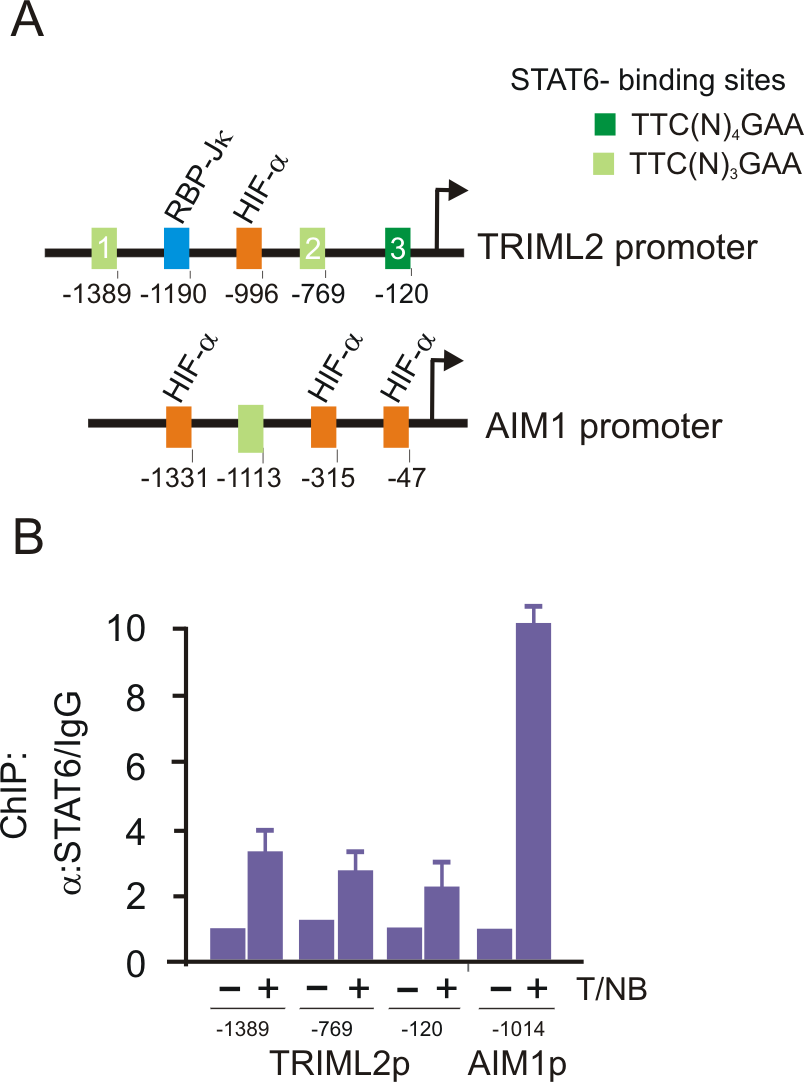

Supplement: S7 Fig — (A) Schematic of putative STAT6, RBP-Jκ and HIFα-binding sites within TRIML2 and AIM1 promoters. (B) STAT6 bound to TRIML2 and AIM1 promoter and enhanced by reactivation of lytic cycle. BCBL1 cells with or without TPA and sodium butyrate (NaB) treatment were subjected to Chromatin immunoprecipitation (ChIP) with endogenous STAT6. Non-specific rabbit IgG were used as control. The relative levels of STAT6 bound to TRIML2 and AIM1 promoter were detected by quantitative PCR, respectively. Data is presented as means±SD of three independent experiments. (TIF) [file ppat.1007416.s008.tif]

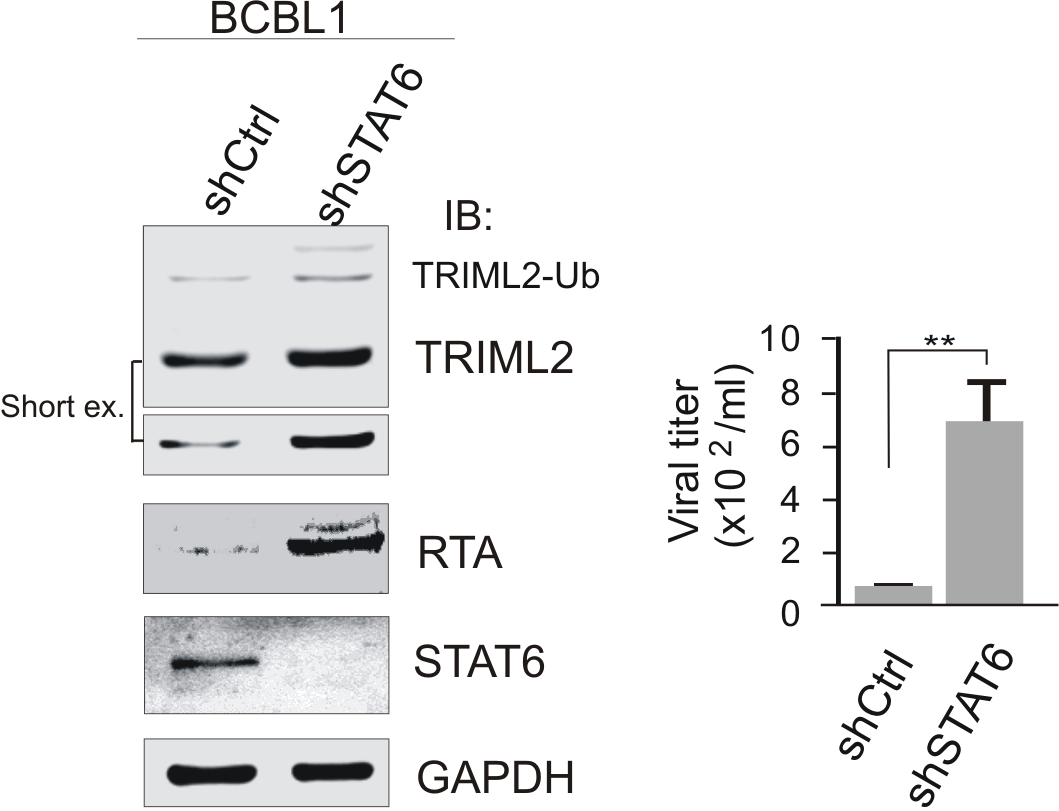

Supplement: S8 Fig — BCBL1 cells were individually infected with lentivirus carrying shSTAT6 or shCtrl control. Equal amounts of knockdown cells were subjected to immunoblotting analysis with antibodies against STAT6, TRIML2 and RTA, and the virion titer in the supernatant of culture media was carried out by quantitative PCR (bottom panel). (TIF) [file ppat.1007416.s009.tif]
