# Supplementary material for: Correction: STAT6 degradation and ubiquitylated TRIML2 are essential for activation of human oncogenic herpesvirus
Source: PLoS Pathog. 2022 Jun 1;18(6):e1010579. doi: 10.1371/journal.ppat.1010579 (PMC9159590; doi:10.1371/journal.ppat.1010579)

Figure 1A

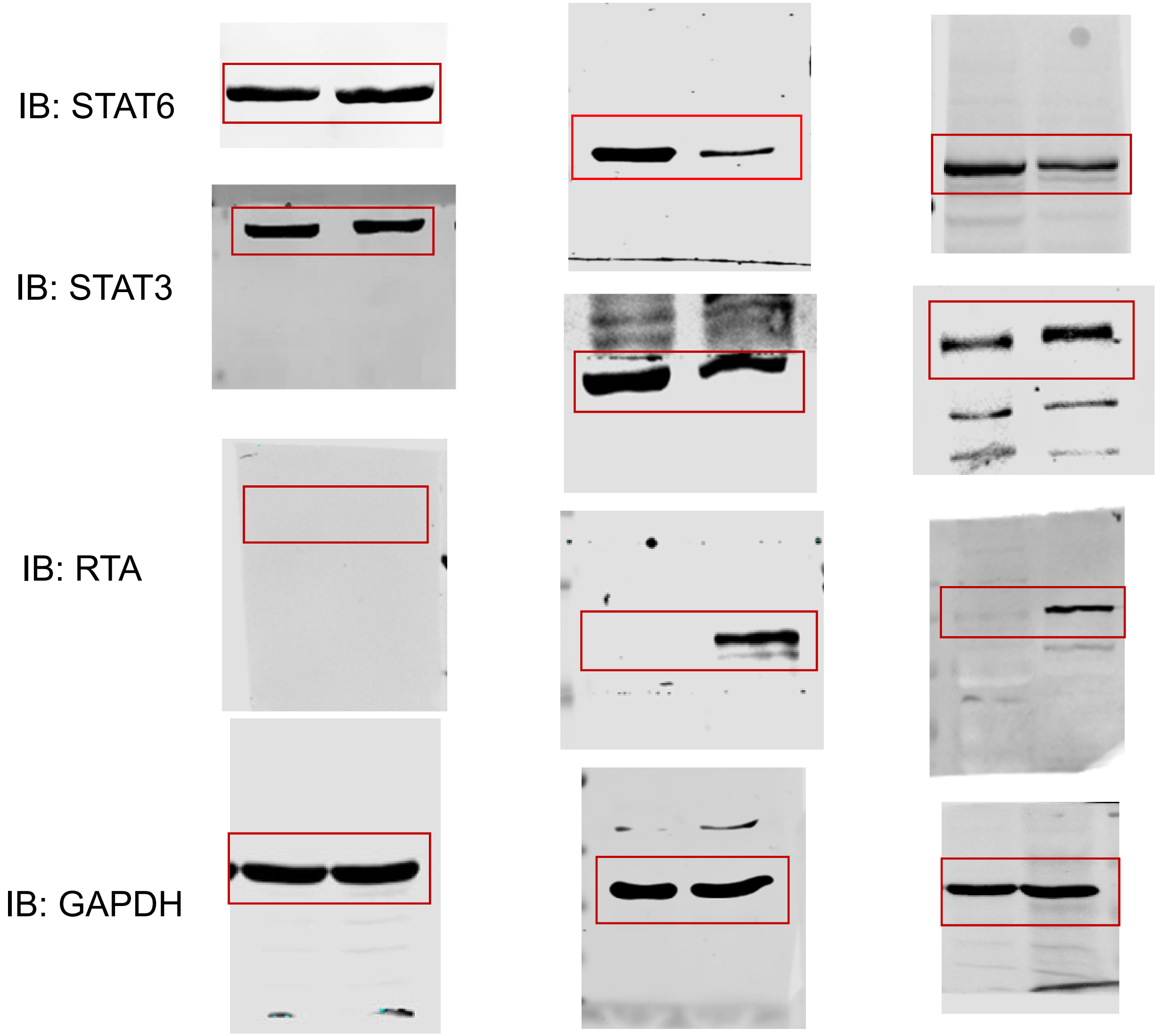

Figure 1B

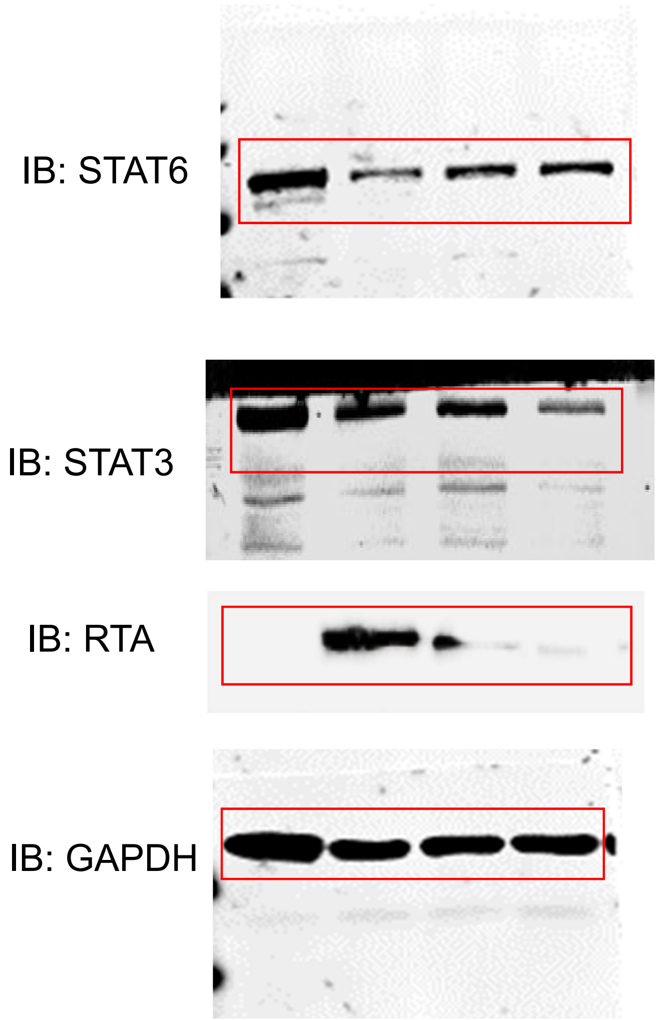

Figure 1C

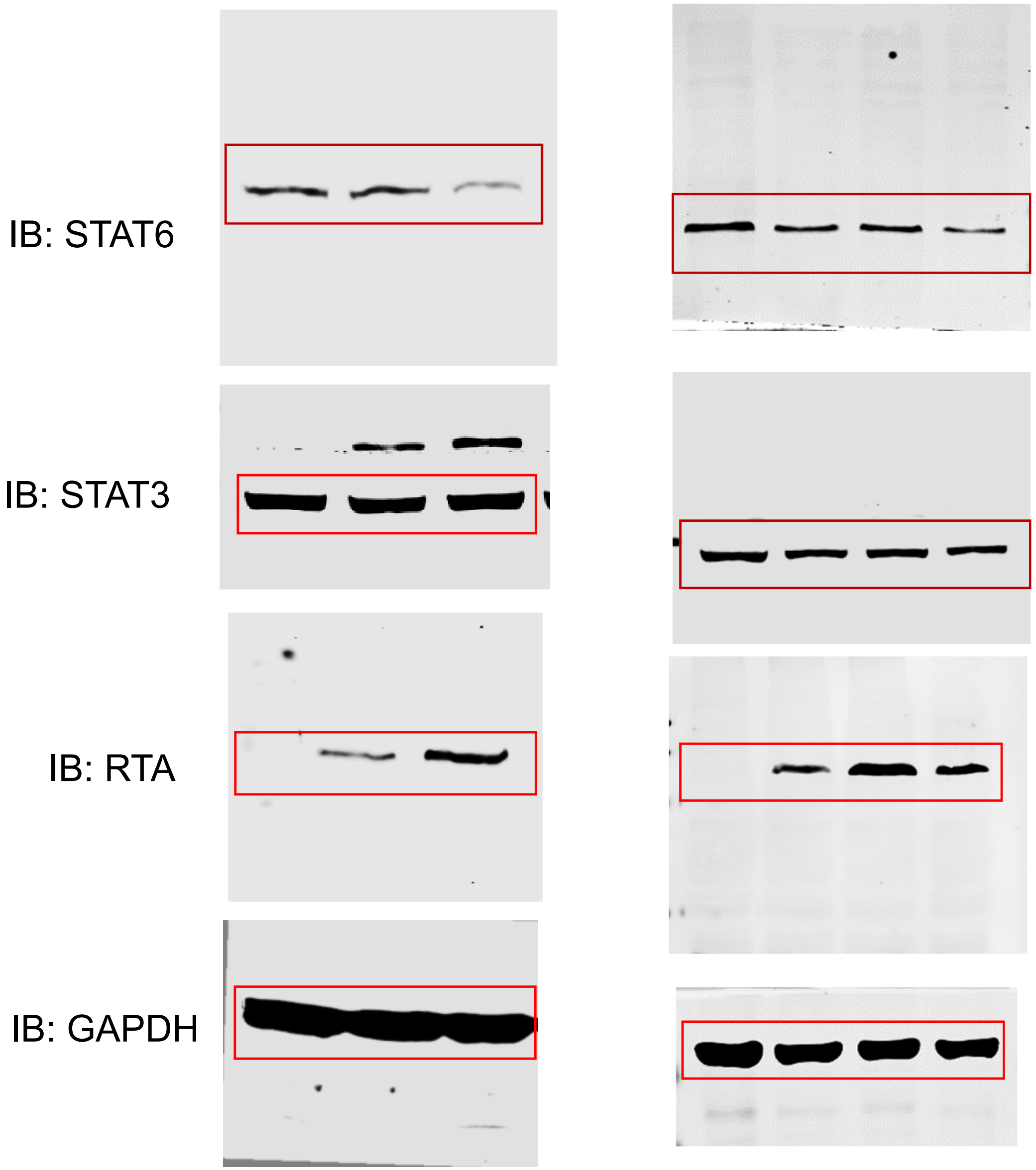

Figure 1F

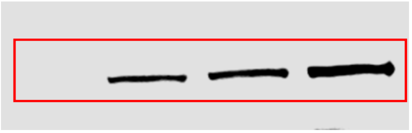

IB: RTA

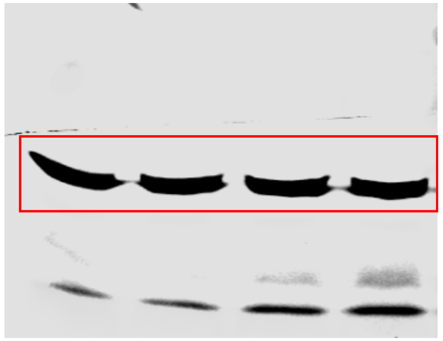

IB: GAPDH

Figure 1G

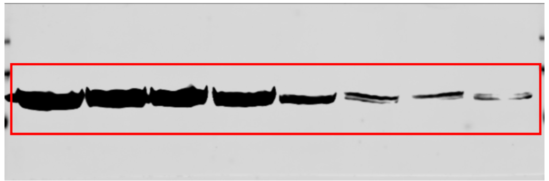

IB: STAT6

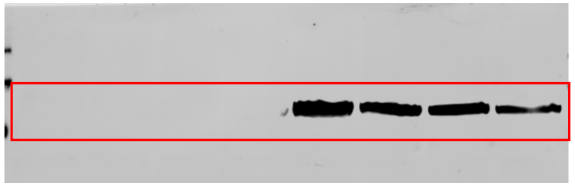

IB: RTA

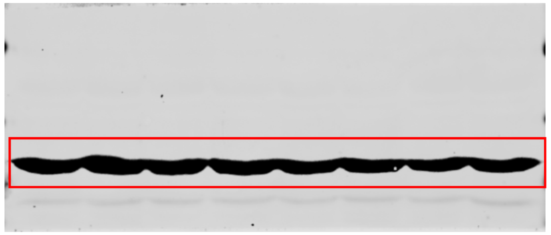

IB: GAPDH

Figure 4A

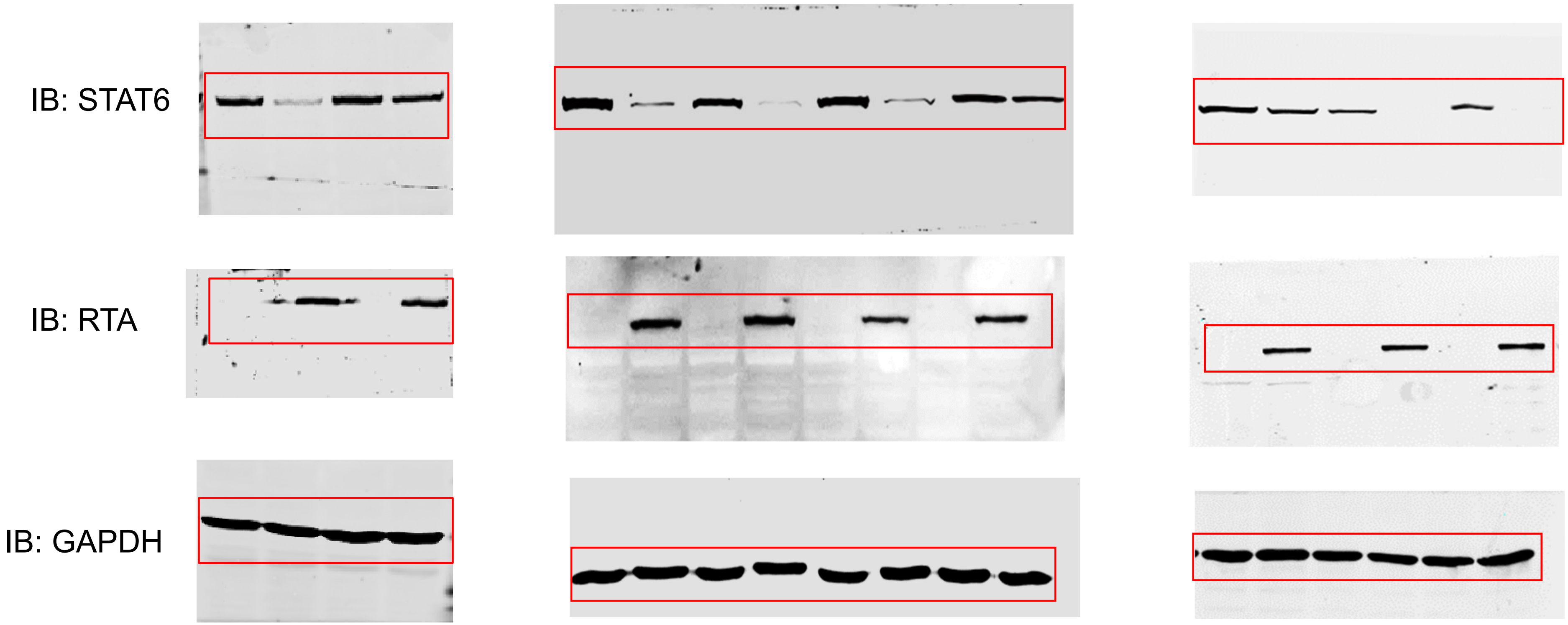

Figure 4B

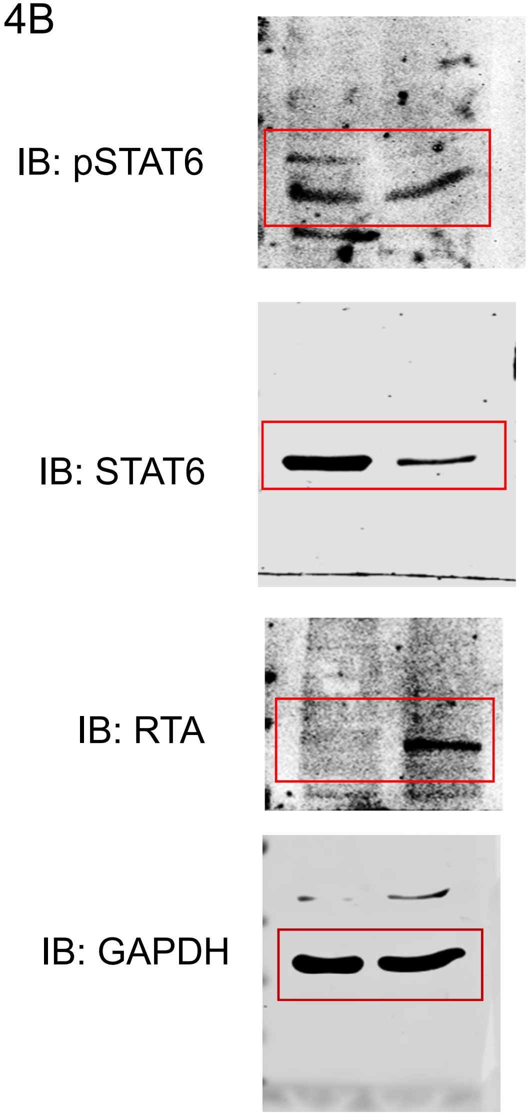

Figure 4C

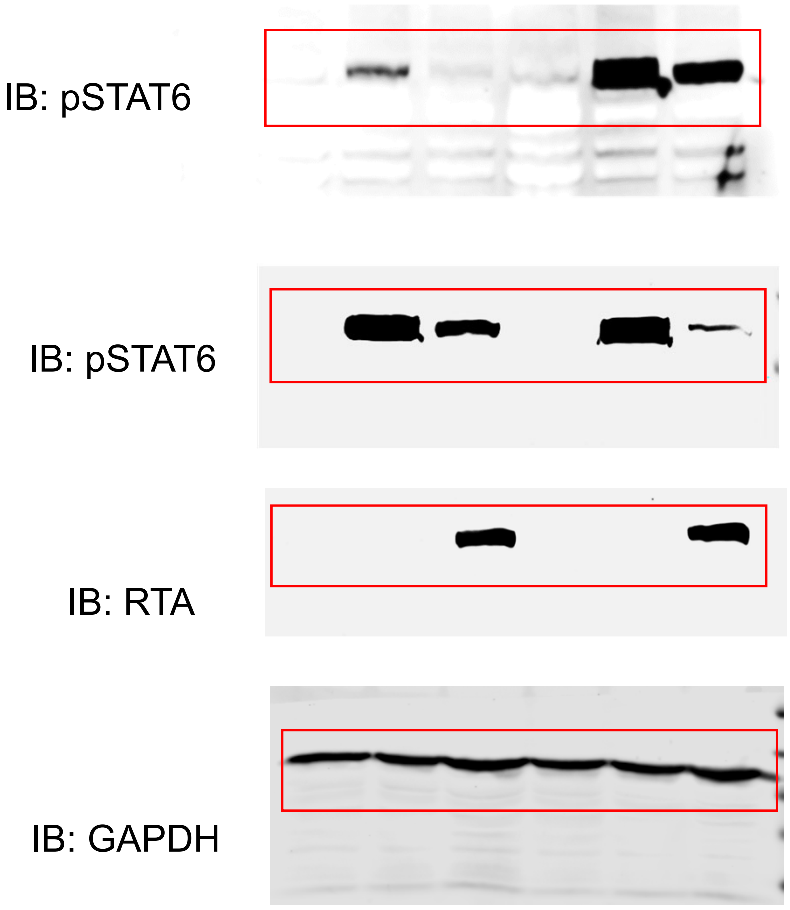

Figure 4D

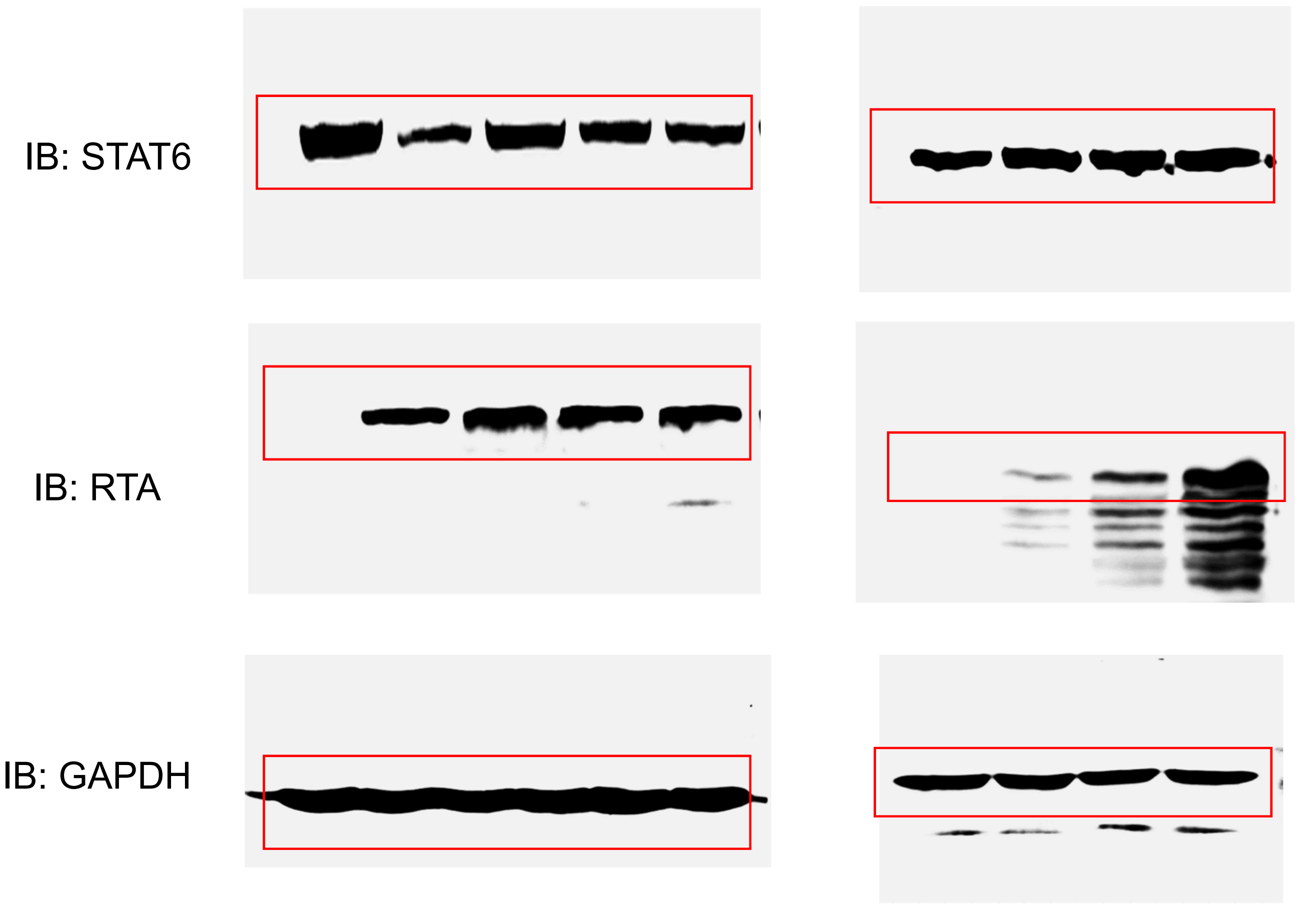

Figure 5A

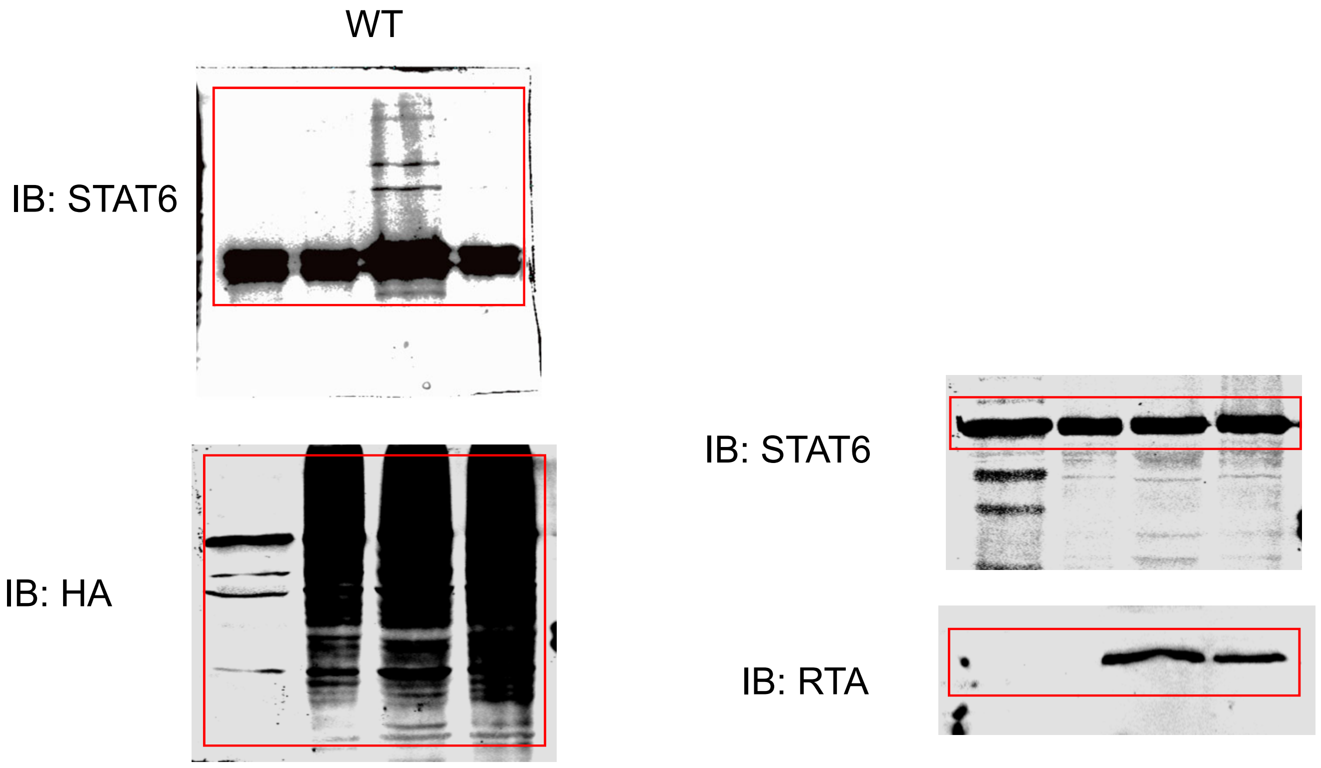

Figure 5B

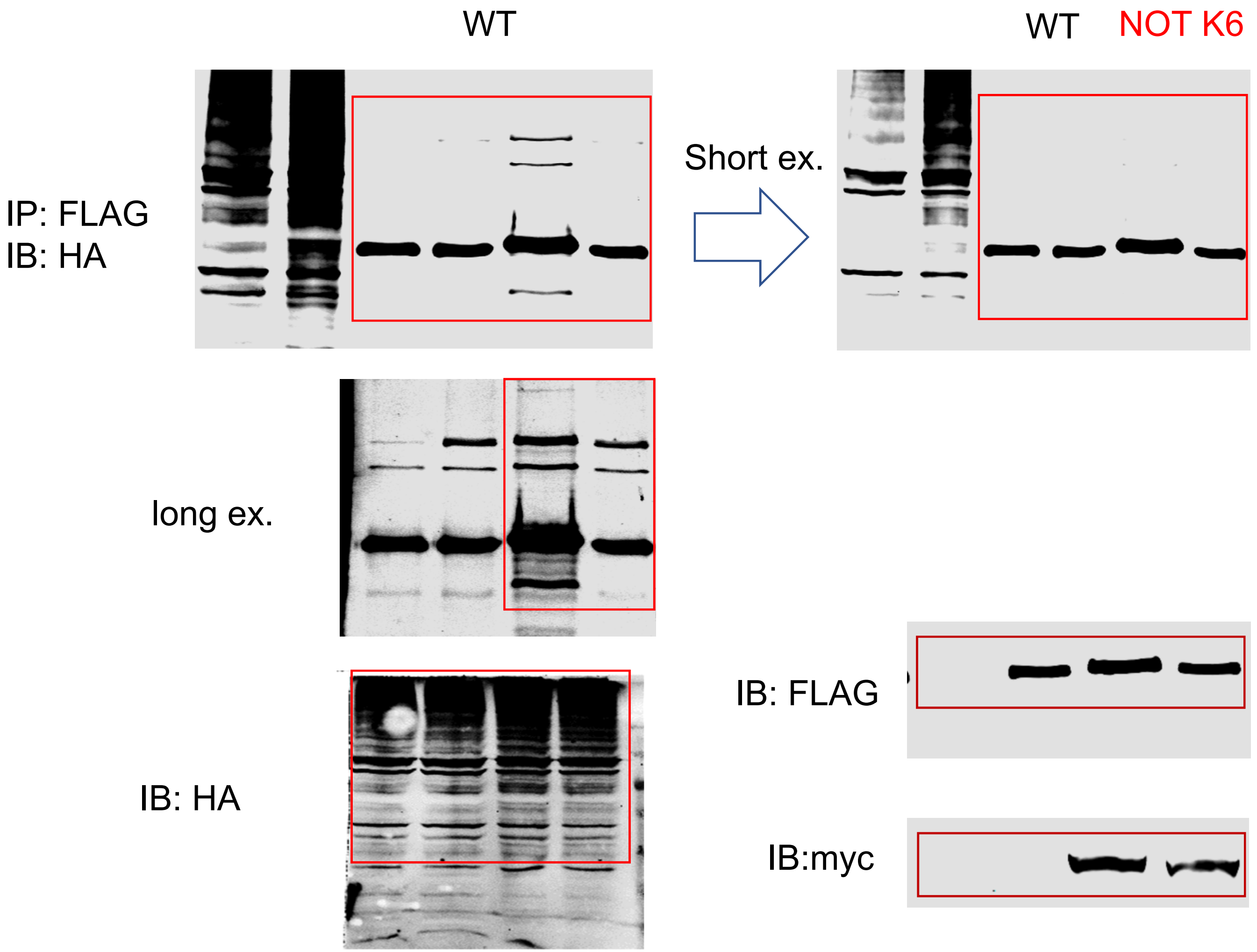

Figure 5B

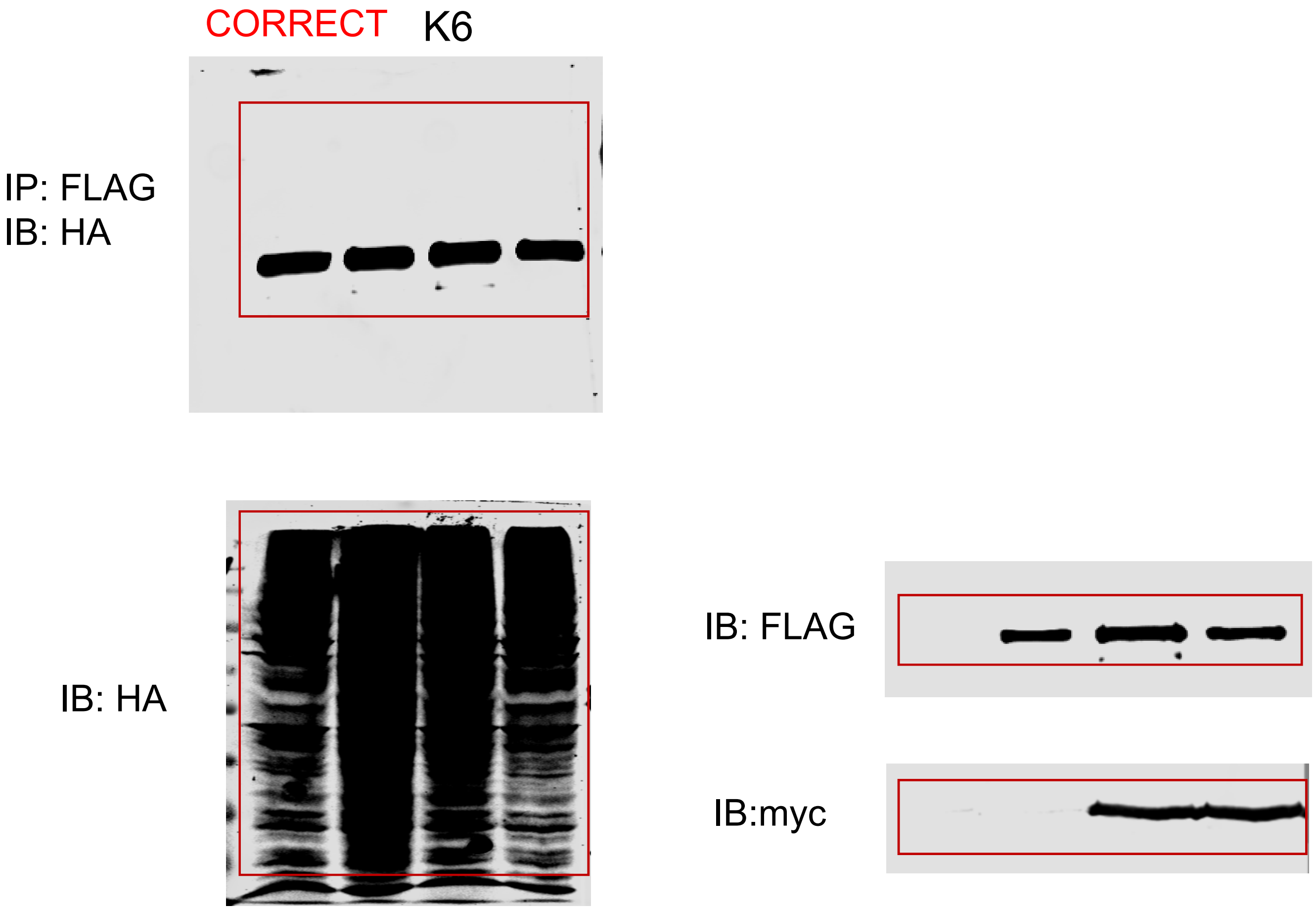

Figure 5B

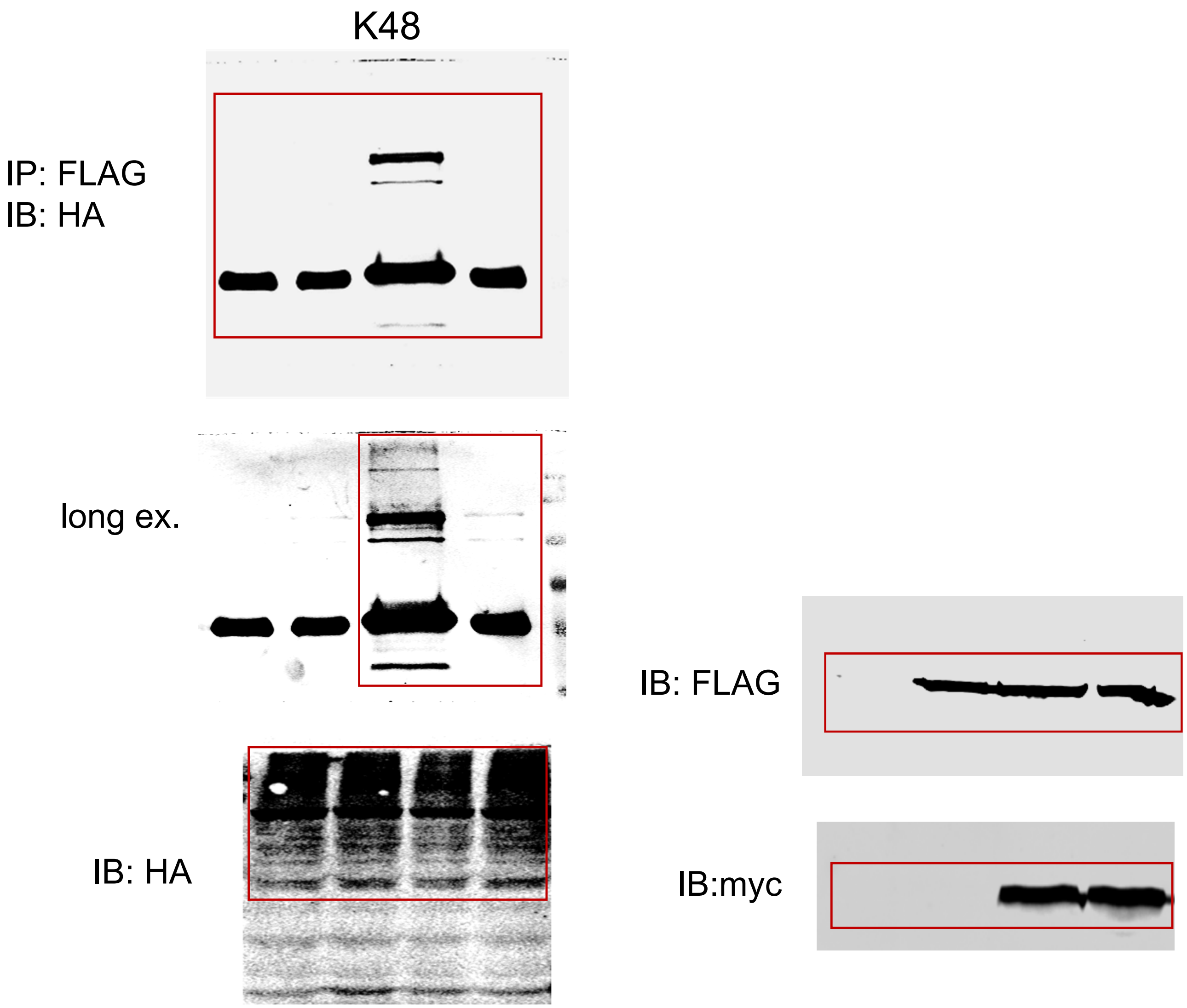

Figure 5B

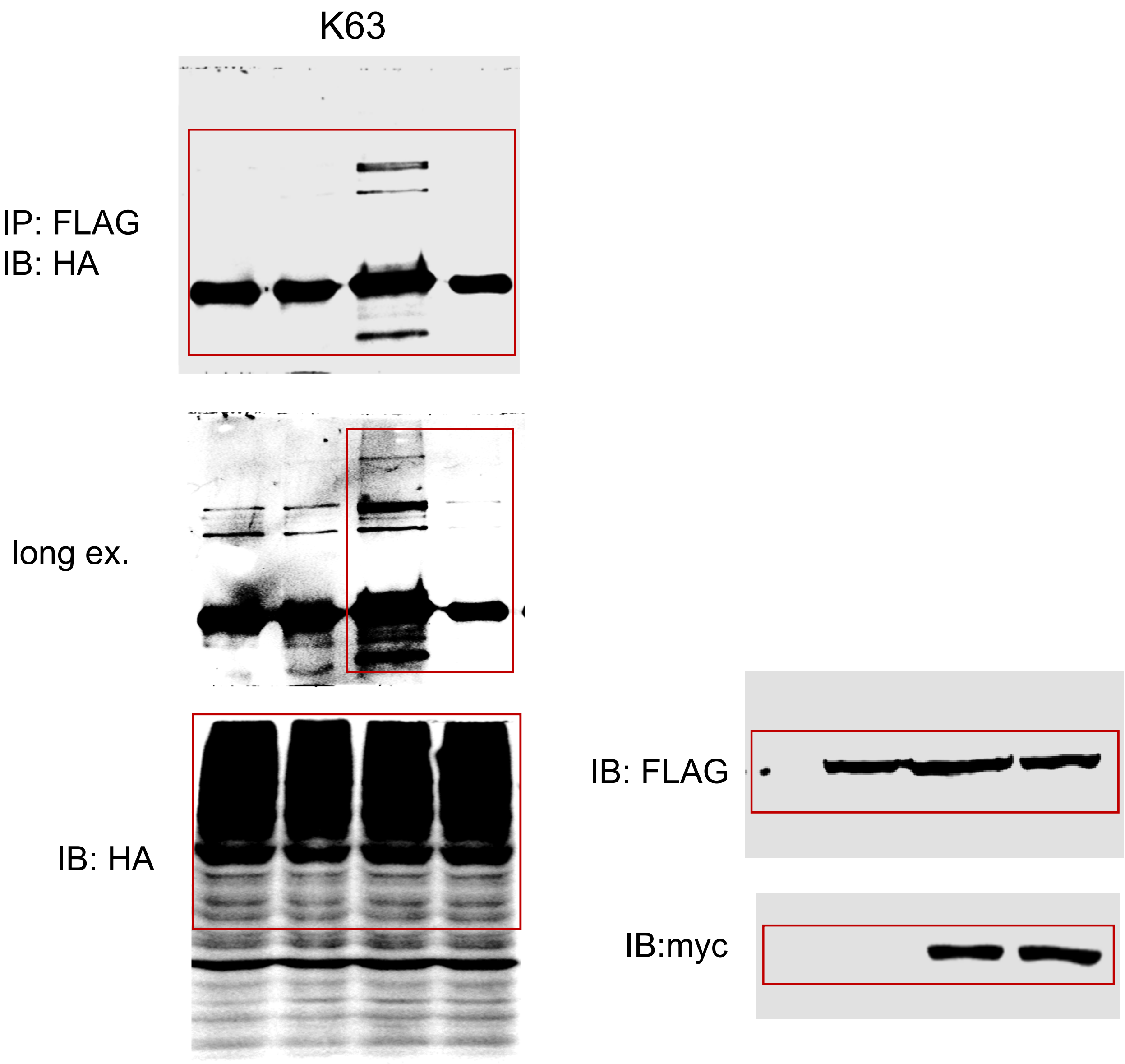

Figure 5C

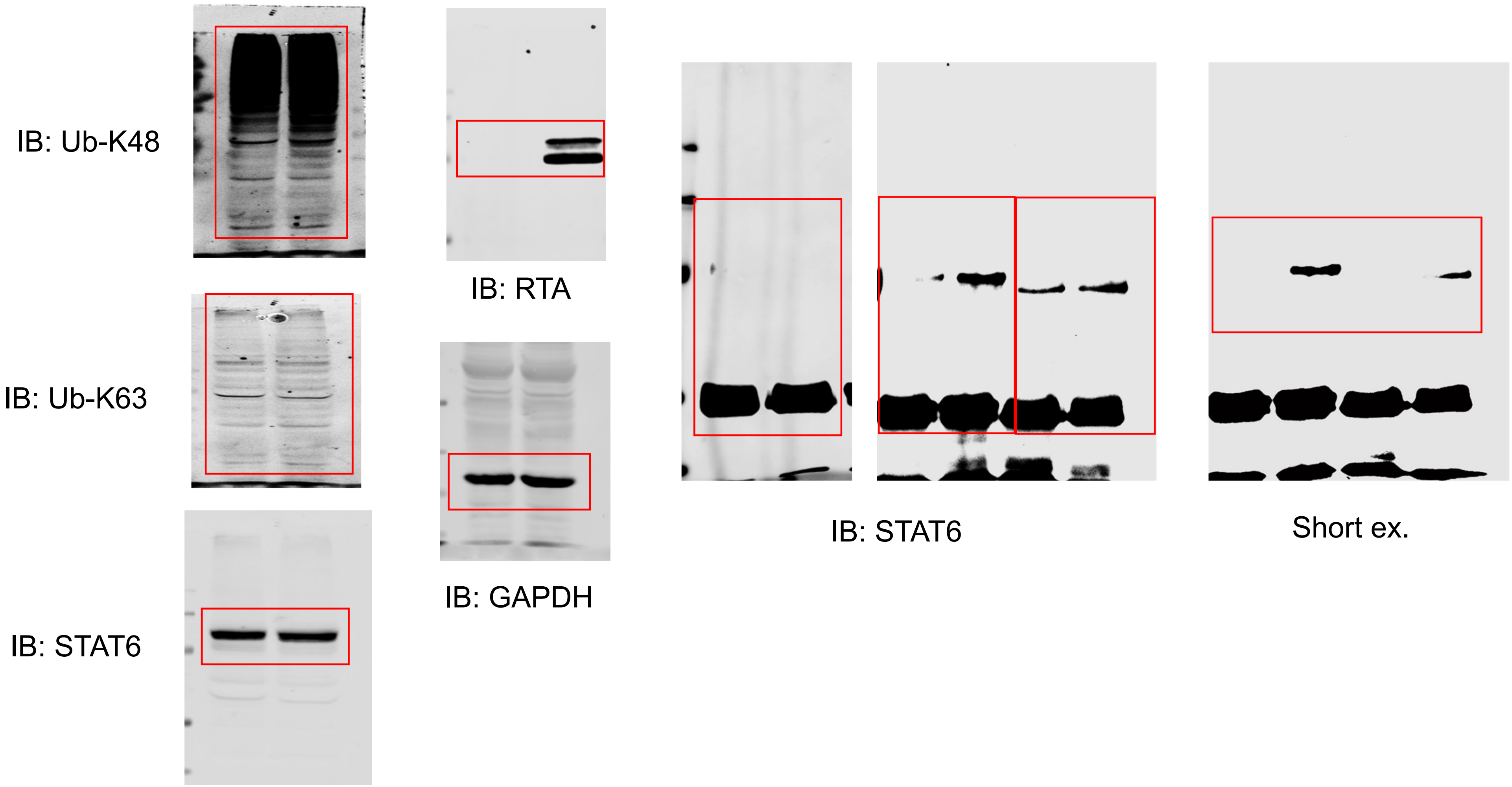

Figure 7A

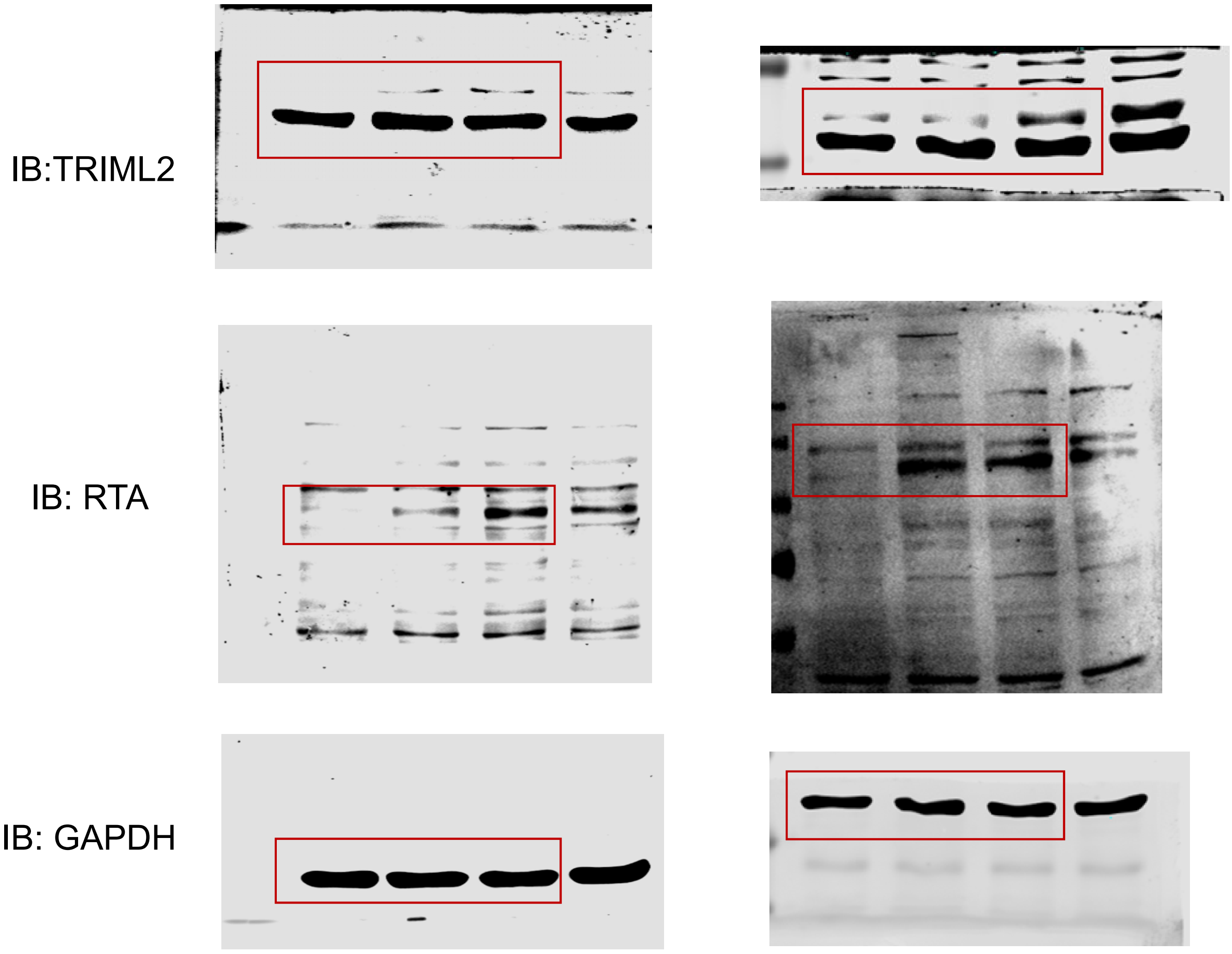

Figure 7B

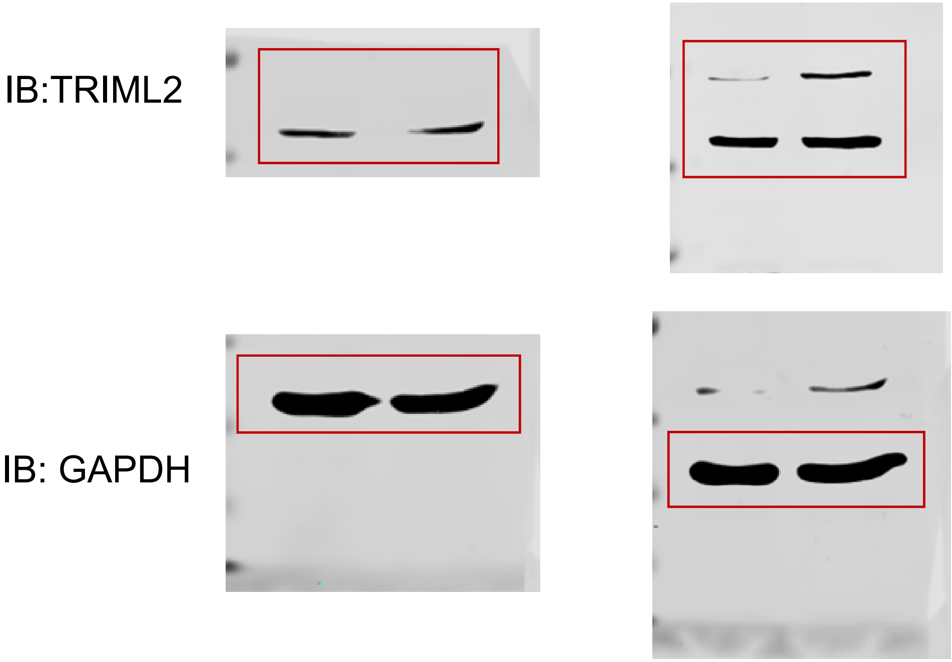

Figure 7C

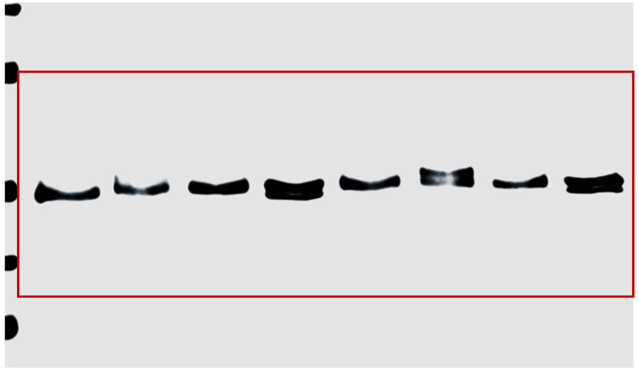

IB:TRIML2

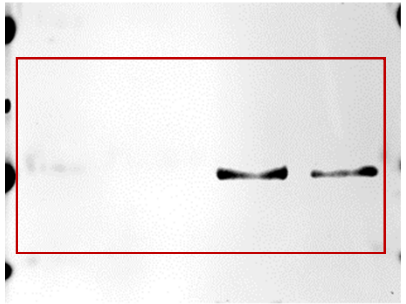

IB:TRIML2

Figure 7D Left

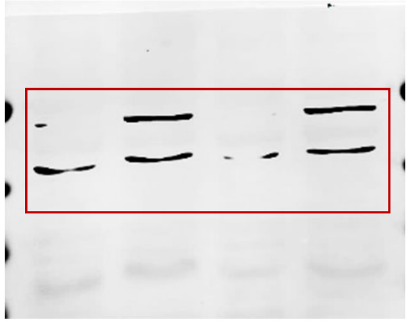
IB:TRIML2

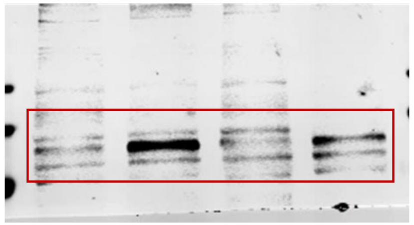
IB:RTA

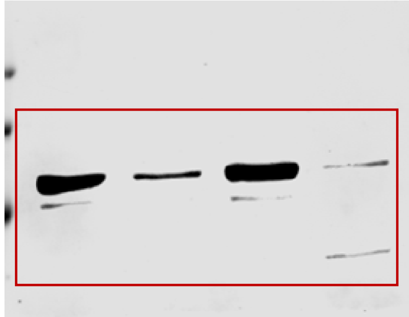
IB:STAT6

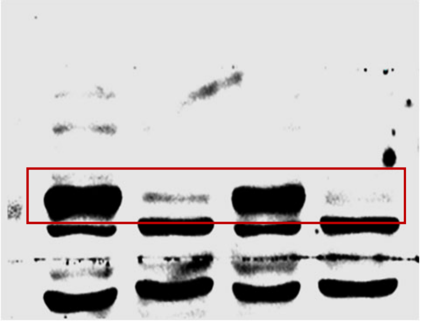
IB: p53

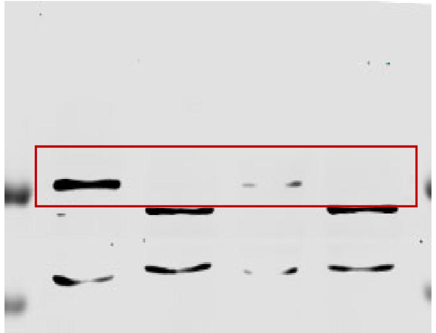
Short ex.

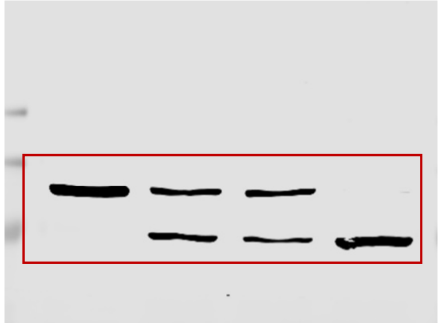
IB: PARP1

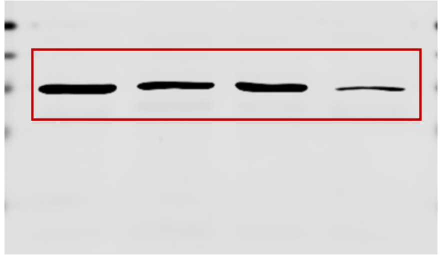
IB: Caspase 3

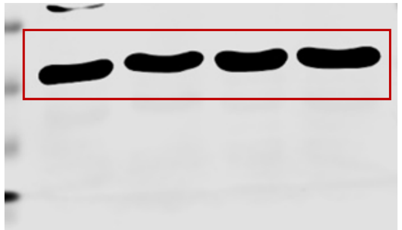
IB: GAPDH

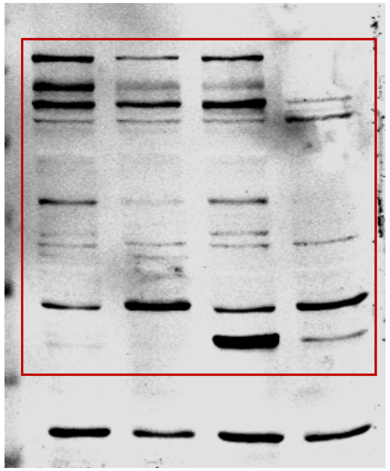
IB: HIF2a

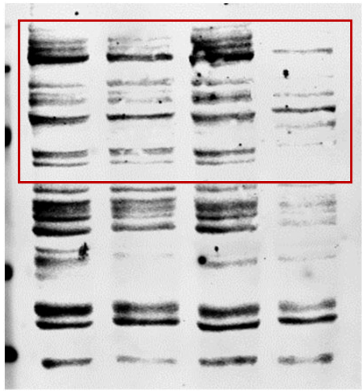
IB: HIF1a

Figure 7D Right

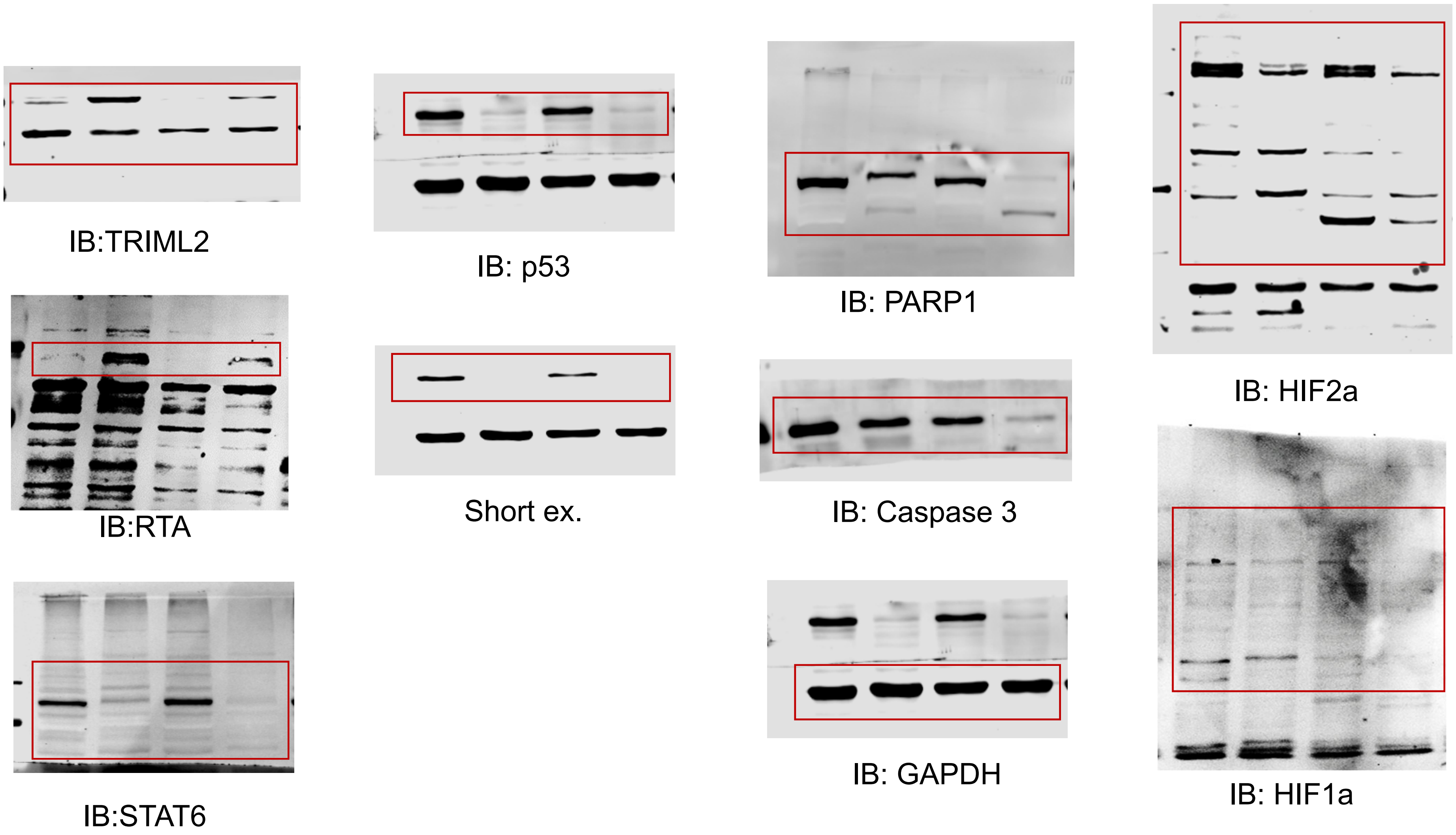

Supplement: S1 File — (PDF) [file ppat.1010579.s001.pdf]
